# Wireless Electroencephalography in Research on Children With Developmental Disabilities: Scoping Review

**DOI:** 10.2196/84707

**Published:** 2026-08-25

**Authors:** Naeun Park, Yoomi Shin, Jaeeun Kang, Young Eun Lee, Anna Lee

**Affiliations:** 1 College of Nursing, Yonsei University, Seoul, Republic of Korea; 2 Lawrence Bloomberg Faculty of Nursing, University of Toronto, Toronto, ON, Canada; 3 Institute for Innovation in Digital Healthcare, Yonsei University, Seoul, Republic of Korea; 4 Mo-Im Kim Nursing Research Institute, College of Nursing, Yonsei University, 50-1, Yonsei-Ro, Seodaemun-gu, Seoul, 03722, Republic of Korea, 82 2-2228-3284

**Keywords:** neurodevelopmental disorder, attention deficit and disruptive behavior disorder, autism spectrum disorder, cerebral palsy, child psychiatry, electroencephalography, brain-computer interface, wireless technology, scoping review

## Abstract

**Background:**

Wireless electroencephalography (EEG) systems offer practical advantages over conventional wired devices in the assessment of children with developmental disabilities (DDs), including enhanced portability, reduced participant burden, and ease of use. However, how these systems have been applied across diverse DD populations, research purposes, and clinical contexts remains unclear.

**Objective:**

This scoping review aimed to map available evidence on wireless EEG applications in children with DDs, characterize device specifications by application purpose, identify neurobehavioral challenges and corresponding methodological solutions, and assess data quality–related reporting practices.

**Methods:**

This scoping review followed the PRISMA-ScR (Preferred Reporting Items for Systematic Reviews and Meta-Analyses Extension for Scoping Reviews), PRISMA-S (PRISMA Statement for Reporting Literature Searches in Systematic Reviews), and the population, concept, and context framework: population, children aged<19 years with DDs; concept, studies using wireless EEG devices for data collection; and context, all research and clinical settings. A systematic search was conducted across 7 databases (PubMed, Embase, IEEE Xplore, Web of Science, CINAHL, PsycINFO, and Scopus) from their inception through December 2025. Screening was performed independently by 4 reviewers. Data on study characteristics, device specifications, neurobehavioral recording challenges, and data quality reporting were extracted and synthesized descriptively, including cross-tabulation of devices by application purpose.

**Results:**

Of 594 identified records, 64 studies enrolling 3103 participants met the inclusion criteria. Studies were published between 2005 and 2025, with an increasing trend in both publications and sample sizes. Attention-deficit/hyperactivity disorder (38/64, 59.4%) and autism spectrum disorder (20/64, 31.3%) were the most frequently studied conditions. Primary application domains were biomarker-driven assessment and diagnosis (33/64, 51.6%), brain-computer interface (BCI) technology (18/64, 28.1%), intervention evaluation (8/64, 12.5%), and task or state monitoring (5/64, 7.8%). Across 65 study-device pairs, consumer-grade devices predominated (31/65, 47.7%), followed by research-use-only (19/65, 29.2%) and medical devices (15/65, 23.1%). Purpose-driven patterns emerged: BCI studies favored low-channel, dry-electrode, consumer-grade devices, whereas biomarker-driven and intervention studies used higher channel counts and greater signal fidelity. Recurring neurobehavioral challenges (eg, inattention, sensory hypersensitivity, and motor impairment) were addressed through rapid, low-preparation electrode setups, child-friendly device designs, and adapted recording protocols such as home-based or caregiver-mediated sessions. Data quality–related reporting was substantially incomplete: 85.9% (55/64) did not report validation against a wired EEG system, 79.7% (51/64) did not specify impedance thresholds, and 12.5% (8/64) described no artifact handling.

**Conclusions:**

This scoping review is the first to comprehensively map wireless EEG research in children across a broad spectrum of DDs—integrating diagnosis, study context, and device characteristics—rather than focusing on a single condition or purpose. This review highlights critical gaps in data quality–related reporting that limit the interpretability and comparability of current findings. Future studies should prioritize rigorous validation within DD cohorts and the development of population-specific guidelines for device selection, signal quality assurance, and reporting transparency.

## Introduction

### Rationale

Developmental disabilities (DDs) are characterized by impairments in cognitive, social, and emotional functioning and encompass conditions such as autism spectrum disorder (ASD), attention-deficit/hyperactivity disorder (ADHD), intellectual disability (ID), and motor developmental disorders [1]. The global prevalence of DDs is increasing significantly [2,3], posing a significant socioeconomic burden worldwide [4]. Early identification and intervention are essential to improve long-term outcomes in this population [5,6]. To achieve such intervention, the underlying neurobiological mechanisms that precede or accompany clinical symptoms with DDs must be elucidated [7].

DDs are frequently associated with atypical brain functioning. For example, children with ASD often exhibit atypical neural connectivity and generalized electroencephalography (EEG) abnormalities, such as the prominent slowing of background theta or delta waves and excessive beta activity [8-10], whereas children with ADHD demonstrate aberrant alpha modulation during attention tasks [11]. Such atypical presentations indicate deficits in the regulation of cortical excitability and selective attention [11]. Recent studies demonstrate the potential of EEG-derived biomarkers as objective, adjunctive tools for early detection. For instance, combining EEG spectral power with eye-tracking metrics within a restricted interest paradigm shows potential in screening young children with ASD, outperforming traditional behavioral assessments alone [12]. Beyond early screening, the clinical applications of EEG in DDs are expanding to include active interventions such as neurofeedback [13].

Despite its significant potential as a high-resolution, noninvasive biomarker tool [14], the application of traditional wired medical-grade EEG systems typically requires lengthy preparation, including gel application and montage selection. Furthermore, wired systems substantially limit participants’ movement and cause considerable discomfort [15-17]. These limitations often reduce compliance and compromise data quality in children with DDs because of limited mobility, heightened sensitivity to unfamiliar environments, and hypersensory responses [16,18]. Consequently, recordings obtained in restrictive, uncomfortable laboratory settings may not accurately represent the child’s authentic brain function during naturalistic social or cognitive activities.

Wireless EEG offers a means of overcoming these challenges. As a mobile, portable, wearable, and ambulatory system, wireless EEG enables both device and participant mobility [19]. Wireless EEG systems, which feature dry electrodes and self-application designs, greatly enhance ease of use and portability [19,20]. These enhancements facilitate the investigation of brain function in naturalistic settings such as home [21], school [22], or workplace [23]. Wireless EEG represents a promising approach for both research and clinical applications involving children with DDs by minimizing challenges commonly observed in this population, such as heightened sensitivity and hypersensory responses. These benefits have led to a surge in basic research using wearable EEGs. However, the results from this vast body of research remain fragmented.

Existing reviews on wireless EEG in this population have predominantly focused on a single purpose (eg, diagnosis [24]) or a specific event (eg, seizure monitoring [25]). However, a comprehensive synthesis evaluating how these technical advancements specifically address the practical hurdles of EEG recording in children with various DDs is lacking. Although Lau-Zhu et al [16] highlighted the potential “opportunities” of mobile EEG for neurodevelopmental disorders through a narrative review, the subsequent surge in research calls for a systematic mapping of the current literature to evaluate its practical utility. This mapping helps in identifying how specific technological advancements address child-specific challenges, such as limited attention spans and sensory hypersensitivities, which uniquely characterize pediatric neurodevelopmental disorders as distinct from typical developmental cases.

### Objectives

To address this gap, this scoping review synthesized current evidence to provide a structured path forward. This scoping review aims (1) to map existing evidence on wireless EEG applications in children with DDs across diverse research contexts and purposes, (2) to identify the most frequently used types of wireless EEG devices and summarize the technical specifications and mobility characteristics of these devices according to their application purposes, (3) to identify the neurobehavioral challenges associated with EEG recording in children with different DDs and map them to appropriate device and methodological approaches, (4) to assess data quality–related reporting characteristics across the reviewed studies, and (5) to identify knowledge gaps and future research directions.

## Methods

This scoping review was conducted in accordance with the PRISMA-ScR (Preferred Reporting Items for Systematic Reviews and Meta-Analyses extension for Scoping Reviews) [26] and the PRISMA-S (PRISMA Statement for Reporting Literature Searches in Systematic Reviews) [27] (Checklist 1).

### Protocol and Registration

A review protocol was not registered in any public registry.

### Eligibility Criteria

The population, concept, and context criteria encompassed children with DDs (population), EEG measurement using a wireless device (concept), and no restrictions (context). Included studies were required to fulfill the following criteria:

- Participants: Children with DDs (age<19 years) with no sex restrictions (eg, ADHD, ASD, ID, or cerebral palsy [CP]). Studies that included both children and adults were excluded.
- Concept: Studies were included if they used wireless EEG devices for data collection. The definition of wireless EEG suggested by Niso et al [19] was adopted in this study, as it characterizes EEG technology systems as those that use wireless protocols for signal acquisition and involve the use of mobile, portable, wearable, or ambulatory EEG devices.
- Context: No restrictions were applied.

Eligible study designs were required to be quantitative and empirical research. Published research protocols, reviews, editorials, books, non-English studies, and non-peer–reviewed studies were excluded. Finally, if the full text could not be obtained, the study was excluded.

### Information Sources

In total, 7 bibliographic databases (PubMed, CINAHL, Web of Science, Embase, PsycINFO, IEEE Xplore, and Scopus) were comprehensively searched from their inception, with no restrictions on publication date. The search was conducted in 2 stages: the first search on February 6, 2025, followed by a second search on January 9, 2026, to include studies published or e-published up to December 31, 2025. No modifications were made to the strategy between searches to capture studies published between the 2 search dates. Each stage was run across all 7 databases to ensure comprehensive coverage of the relevant literature.

### Search

The initial search terms were adapted from prior reviews [28] and tailored to our population, concept, and context. The final search strategy, developed in collaboration with library specialists, included three categories: (1) wireless EEG, (2) child, and (3) DDs. The full electronic search strategy for each database is provided in Multimedia Appendix 1. Published search filters and database-level study design filters were not applied, and only English-language studies were included during study selection. Multidatabase searching was not applicable because each database was searched separately rather than concurrently via a single platform. In addition, no study registries or additional online or print sources were searched for this review.

To supplement the database search, backward citation searching was performed by manually screening the reference lists of included papers. No authors, experts, manufacturers, or other individuals were contacted, and no additional information sources or search methods were used beyond database searching and backward citation searching.

### Selection of Sources of Evidence

The retrieved records were imported into Rayyan, a web-based systematic review tool for screening and duplicate removal [29]. Titles and abstracts were screened independently by 4 reviewers (NP, YS, JK, and YEL), and each record was assessed by 2 reviewers. Prior to formal screening, a calibration exercise was conducted in which all 4 reviewers (NP, YS, JK, and YEL) independently screened a random sample of 10 records to ensure consistent application of the eligibility criteria. Full-text papers of potentially eligible records were then independently screened by the same 4 reviewers, with each paper assessed by 2 reviewers. At both stages, discrepancies were resolved through discussion and consensus during regular research meetings.

### Data Charting and Items

A standardized data charting form was developed in Microsoft Excel. Prior to formal charting, all 4 reviewers (NP, YS, JK, and YEL) jointly charted the same pilot paper to align definitions and interpretation of each field. After alignment, the included studies were divided among the 4 reviewers for independent charting; each reviewer’s charted set was subsequently cross-checked by the other reviewers (each reviewer rereviewed papers other than those they had originally charted), and any discrepancies were resolved by team discussion. The data charting form was iteratively refined during the review process; for example, device-specific fields such as regulatory status and approximate cost range were added after reviewing the full texts.

The following data were extracted from each included study: (1) study characteristics, (2) EEG device technical specifications, and (3) data quality–related reporting. Study characteristics included the first author, year of publication, country of study, EEG recording environment, study design, study objective, EEG data analysis, main findings, participant characteristics (type of DDs, sample size, and age), EEG application purpose, specific role of EEG, and EEG recording-related challenges. EEG application purpose refers to the broad research objective for which wireless EEG was used; it was initially recorded as free text and subsequently synthesized into 4 categories after all charting was completed. Specific role of EEG data refers to the function that EEG data served within each application purpose (eg, predictive modeling input or part of polysomnography).

Technical specifications included the wireless EEG device model and manufacturer, wireless transmission method, sampling rate, number of channels, electrode type, device design, and mobile EEG device category according to the Categorization of Mobile EEG Devices (CoME-D) taxonomy [30]. Devices were also classified by regulatory status into 3 categories: medical devices (cleared or certified by a national regulatory body, including the US Food and Drug Administration, Conformité Européenne marking, or Korean Ministry of Food and Drug Safety), consumer-grade devices (commercially available products not classified as medical devices), and research-use-only (RUO) devices (intended solely for investigational purposes).

Data quality–related reporting included artifact handling methods, impedance thresholds, and validation against a wired EEG system. For each domain, reporting completeness was classified into 3 tiers: not reported, reported with a limited or opaque method (eg, reliance on indirect validation or proprietary or nontransparent artifact processing), and reported with a transparent method (eg, direct validation and use of established signal processing techniques such as independent component analysis [ICA] or artifact subspace reconstruction [ASR]).

### Synthesis of Results

This review used the analytical procedures for scoping reviews outlined in the frameworks of Arksey and O’Malley [31] and Peters et al [32]. Given the substantial heterogeneity across the included studies, a descriptive analysis approach was adopted. Frequencies, percentages, medians, and IQR were used to summarize study characteristics and device specifications; cross-tabulations were performed to examine patterns in device selection across application purposes. An evidence gap map was constructed to visualize the distribution of study-diagnosis pairs across application purposes and DD categories, using EPPI-Mapper (version 2.4.5; EPPI-Centre). Neurobehavioral challenges associated with EEG recording were narratively synthesized and mapped to corresponding device and methodological approaches. Data quality–related reporting completeness was visualized using stacked bar charts. Consistent with scoping review methodology [32], a formal critical appraisal (risk-of-bias assessment) of individual sources of evidence was not performed, as the purpose of this review was to map the breadth and characteristics of the existing literature.

## Results

### Selection of Sources of Evidence

**Figure 1.**
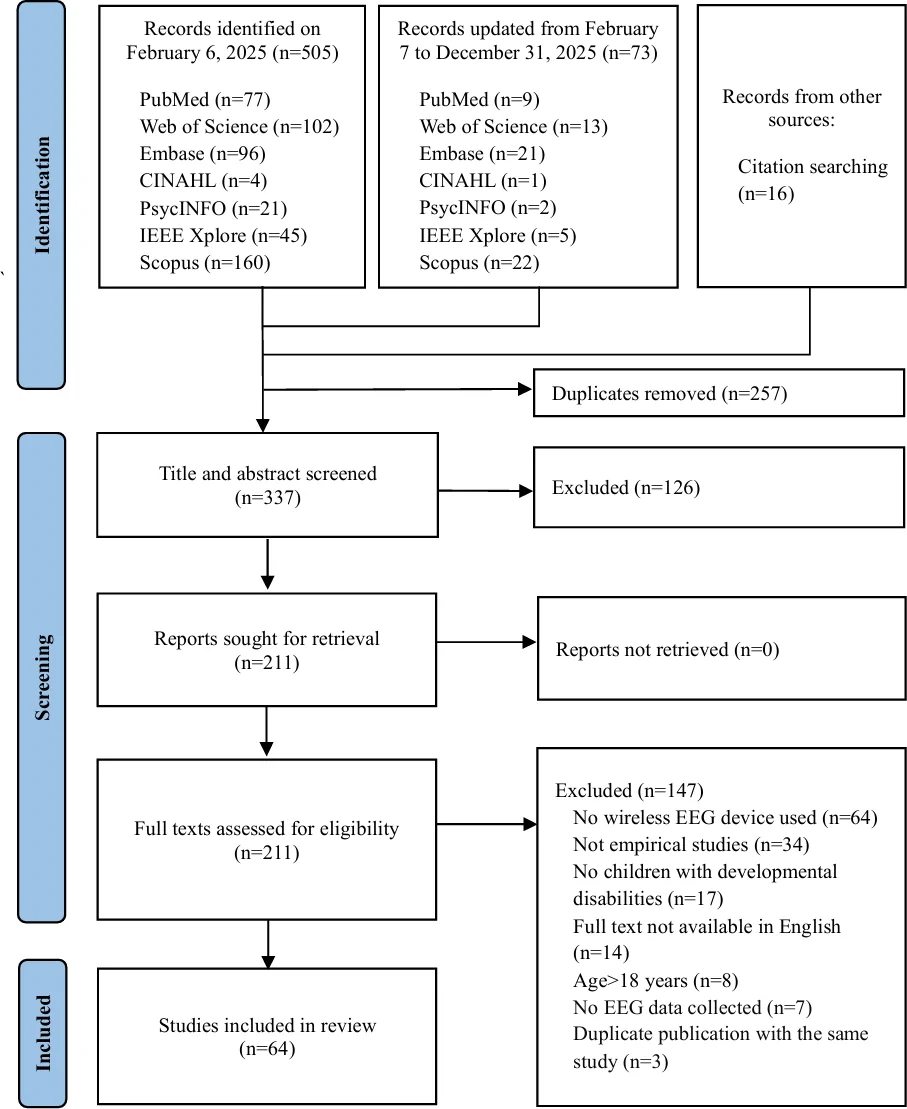
PRISMA-ScR (Preferred Reporting Items for Systematic Reviews and Meta-Analyses Extension for Scoping Reviews) flowchart. EEG: electroencephalography.

Overall, 594 publications were initially identified. After duplicate removal, 337 records were subjected to title and abstract screening, resulting in 211 papers for full-text review. Among these, 64 studies were included in the final review (Figure 1).

### Characteristics of Sources of Evidence

**Figure 2.**
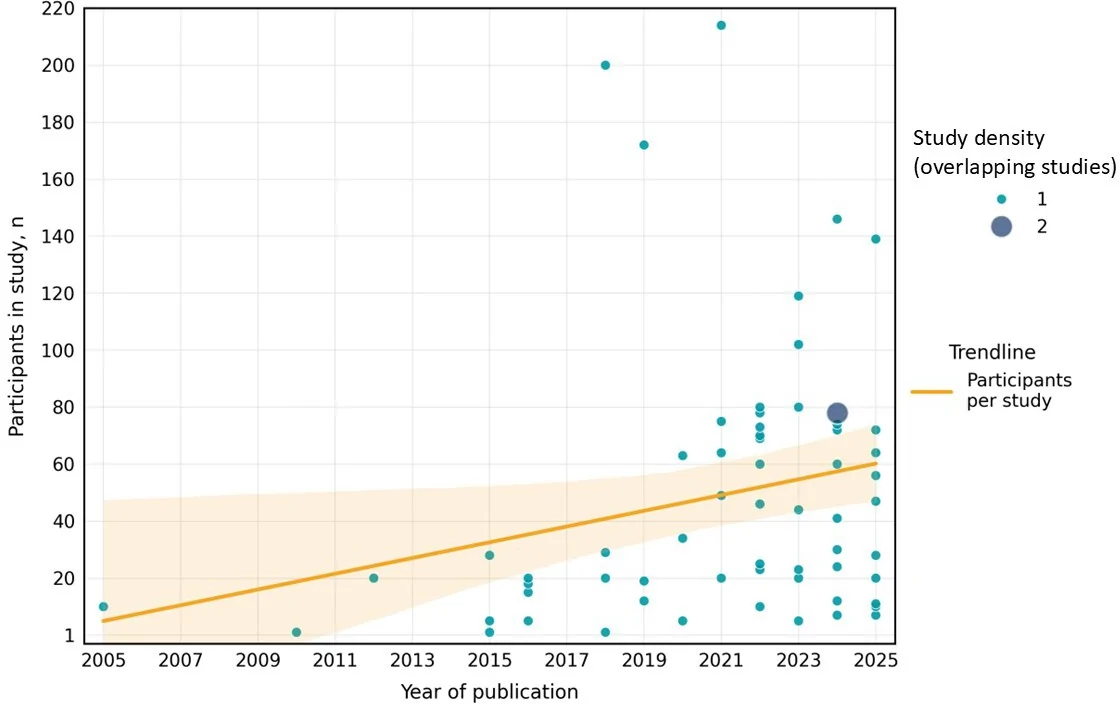
Temporal trends in publication and participant enrollment (2005‐2025). The scatter plot illustrates the number of participants in each study over time. The size of the circles indicates the number of overlapping studies within a given year. The orange trend line illustrates the average growth in participants per study, whereas the shaded areas represent the CI.

The 64 included studies enrolled 3103 participants and were published or e-published between September 2005 and December 2025 [33-96]. Overall, the number of publications and study sample sizes increased over time (Figure 2). The largest sample size was observed in 2021 (n=214) in a study conducted in China [83].

Of the included studies, 41 (64.1%) used an observational design, whereas the remainder adopted an experimental design (23/64, 35.9%; Table 1). Most studies were conducted in Asia (43/64, 67.2%), and Taiwan (15/64, 23.4%) and China (11/64, 17.2%) were the most frequently represented countries. Clinical or laboratory settings were the most frequently reported EEG recording environments (42/64, 65.6%), followed by naturalistic environments such as homes and schools (15/64, 23.4%). The remaining studies did not specify the recording environment (7/64, 10.9%).

**Table 1.** Characteristics of the included studies (N=64).

Study characteristics	Values, n (%)
Study design	64 (100)
Observational study^a^	41 (64.1)
Experimental study (randomized controlled trial)	16 (25)
Experimental study (nonrandomized controlled trial)	7 (10.9)
Continents	64 (100)
Asia	43 (67.2)
North America	9 (14.1)
Europe	9 (14.1)
South America	2 (3.1)
Oceania	1 (1.6)
EEG^b^ recording environment	64 (100)
Clinical (or laboratory)	42 (65.6)
Home	6 (9.4)
Home and clinical (or laboratory)	4 (6.3)
School	4 (6.3)
School and clinical	1 (1.6)
Not specified	7 (10.9)
Participant type^c^	68 (106.3)
ADHD^d^	38 (59.4)
ASD^e^	20 (31.3)
CP^f^ or ISCP^g^	3 (4.7)
ID^h^	3 (4.7)
LD^i^ or SLD^j^	3 (4.7)
OCD^k^	1 (1.6)
Age group^c^	95 (148.4)
Toddlers (1‐2 years)	2 (3.1)
Preschoolers (3‐5 years)	17 (26.6)
School-aged (6‐12 years)	56 (87.5)
Adolescents (13‐18 years)	20 (31.3)
EEG features extracted^c^	80 (125)
Frequency domain (eg, PSD^l^)	40 (62.5)
Time domain (eg, ERP^m^)	12 (18.8)
Connectivity (eg, coherence)	9 (14.1)
Proprietary index	8 (12.5)
Time-frequency domain (eg, wavelet transform)	6 (9.4)
Complexity (eg, entropy)	5 (7.8)

a Observational studies included cross-sectional, case study, methodological, validation, feasibility pilot, and program evaluation studies.

b EEG: electroencephalography.

c Studies were allowed to be classified into more than 1 category for participant type, age group, and EEG features extracted if multiple characteristics were applicable; therefore, percentages may exceed 100%.

d ADHD: attention-deficit/hyperactivity disorder.

e ASD: autism spectrum disorder.

f CP: cerebral palsy.

g ISCP: infantile spastic cerebral palsy.

h ID: intellectual disability.

i LD: learning disorder.

j SLD: specific learning disorder.

k OCD: obsessive-compulsive disorder.

l PSD: power spectral density.

m ERP: event-related potential.

ADHD (38/64, 59.4%) was the most frequently studied condition, followed by ASD (20/64, 31.3%). Several studies enrolled heterogeneous groups comprising children with different types of DDs, such as ADHD and learning disorder (LD), within a single study [44]. In these cases, each diagnosis was recorded separately for classification.

Participants were between 1 and 18 years of age. For the studies that included broad age ranges, participants were classified by developmental stage. School-aged children (6‐12 years) were the most frequently studied group (56/64, 87.5%), whereas toddlers were the least represented group (2/64, 3.1%).

The most commonly extracted EEG features were in the frequency domain, such as power spectral density (40/64, 62.5%), followed by the time domain, including event-related potentials (ERPs; 12/64, 18.8%), connectivity measures, such as coherence (9/64, 14.1%), and proprietary indices (8/64, 12.5%). Several studies extracted features from more than 1 domain. Full study details are provided in Multimedia Appendix 2 [33-96].

### Results of Individual Sources of Evidence I: Application Purposes of Wireless EEG Systems

#### Overview

**Figure 3.**
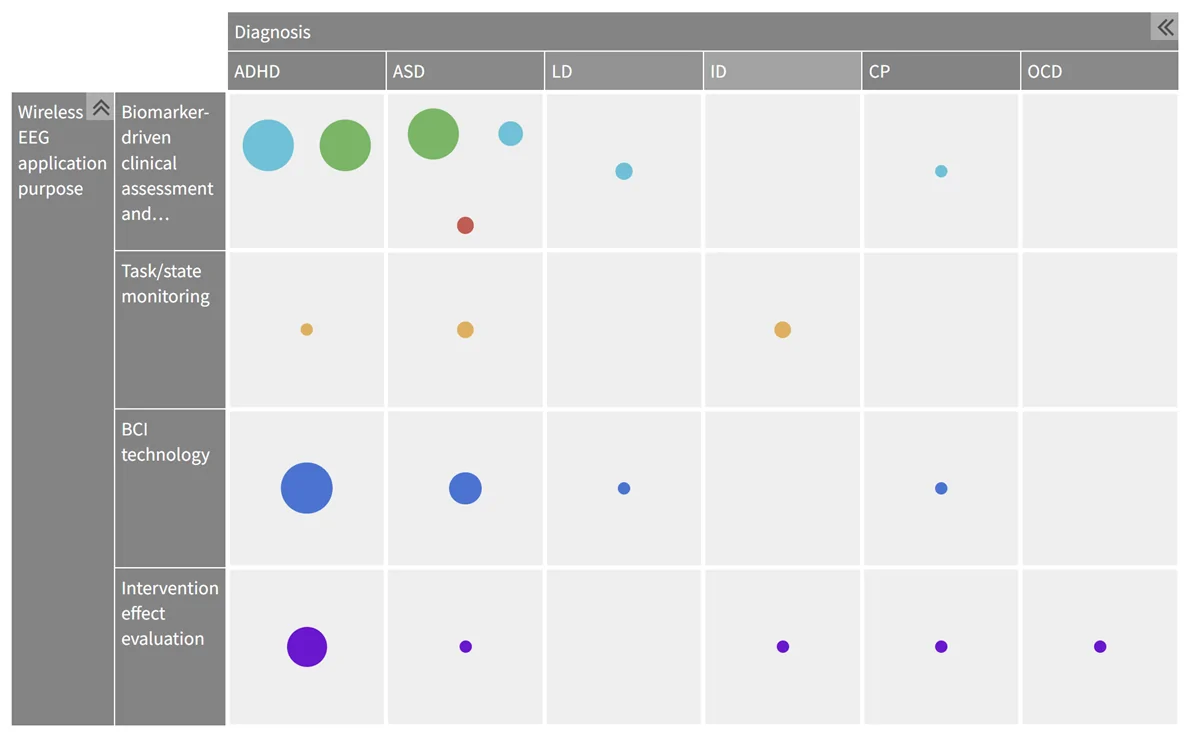
Evidence gap map of wireless EEG research in children with developmental disabilities. The map displays the distribution of 68 study-diagnosis pairs from 64 included studies according to wireless EEG application purpose (rows) and developmental disability (columns). Each cell contains the number of studies corresponding to that combination. Within the cells, studies are segmented by the specific functional role of EEG data, each displayed in a different color: input for machine learning and classification (green), neurophysiological metric (light blue), part of polysomnography (red), real-time state monitor (orange), neurofeedback or assistive technology (dark blue), and outcome measure (purple). The size of each circle is proportional to the number of studies. Generated using EPPI-Mapper (version 2.4.5) powered by EPPI-Reviewer; the interactive version is available at Zenodo [97]. ADHD: attention-deficit/hyperactivity disorder; ASD: autism spectrum disorder; BCI: brain-computer interface; CP: cerebral palsy; EEG: electroencephalography; ID: intellectual disability; LD: learning disorder; OCD: obsessive-compulsive disorder.

The included studies were mainly categorized into four groups according to the primary use of the wireless EEG system: (1) biomarker-driven clinical assessment and diagnosis (33/64, 51.6%), (2) brain-computer interface (BCI) technology (18/64, 28.1%), (3) intervention effect evaluation (8/64, 12.5%), and (4) task or state monitoring (5/64, 7.8%). Figure 3 presents the evidence gap map of the 68 study-diagnosis pairs across application purposes and DD categories. In total, 4 studies contributed 2 diagnosis pairs each due to multiple or co-occurring diagnoses [42,44,45,82]. Evidence was heavily concentrated in ADHD and ASD, which together accounted for 85% of all pairs, whereas ID, LD, CP, and obsessive-compulsive disorder (OCD) were each represented by 3 or fewer pairs, with notable gaps in biomarker-driven and BCI research for these populations.

#### Biomarker-Driven Clinical Assessment and Diagnosis

Studies aiming to identify and use neurophysiological EEG biomarkers for objective assessment, screening, or diagnostic support of DDs were classified in this category. Most of these studies involved children with ADHD (n=19), followed by those with ASD (n=13), LD (n=2), and CP (n=1; Figure 3).

EEG data were most frequently used as primary inputs for predictive modeling (n=17). To classify children with DDs (ADHD: n=9; ASD: n=8) from typically developing peers, the majority of studies (n=15) used machine learning techniques. These included traditional machine learning models, predominantly using support vector machines [40,47,55,59,67,69,72,84,88,91-93], as well as advanced deep-learning architectures [50,64,96]. Additionally, the remaining studies (n=2) used statistical classification approaches, such as discriminant function analysis and multimodal fusion classifiers [83,90].

In total, 14 studies focused on identifying atypical neurophysiological signatures associated with DDs. Among ADHD studies (n=10), distinct spectral features related to cognitive and attentional deficits were consistently reported [33,43,44,46,51,52,63], including prominent delta synchronization during sustained attention tasks [46]. Additionally, atypical ERPs—such as delayed P300 latencies—were observed during auditory oddball paradigms [42]. Of these, 2 studies also included children with LD, applying the same spectral analysis approach to both populations [42,44]. In ASD studies (n=3), lower brain signal complexity in frontal and temporal regions [66] and attenuated P3a and late positive potential amplitudes with reduced theta and alpha connectivity during social tasks were reported [53,70]. A study on children with CP indicated generally lower EEG power and a higher theta/beta ratio [39].

In total, 2 studies investigated sleep parameters related to core ASD symptoms using a polysomnography device [62,94].

#### BCI Technology

Studies applying BCI technology, which involved recording real-time EEG signals and translating neural patterns into control commands, were categorized into this domain. Most involved children with ADHD (n=13), followed by ASD (n=4), CP (n=1), and LD (n=1; Figure 3).

In total, 17 studies used neurofeedback systems, a BCI application that provides real-time feedback on brain activity to facilitate neural self-regulation [98]. Most targeted children with ADHD (n=13), primarily to enhance cognitive and attentional functions [35,48,49,60,75,76,78,81,82,85-87,95]. Recent studies have demonstrated the feasibility of home-based neurofeedback training, with improvements in inattentive symptoms and executive functions comparable with clinic-based interventions [60,85]. For children with ASD (n=4), neurofeedback training focused on improving social communication and interaction skills [61,71,80,82], including gamified approaches using mobile augmented reality [61]. A study evaluated a smartphone-based neurofeedback app for children with dyslexia (LD) [41].

A study implemented a BCI system as “assistive technology” for children with CP and severe motor disabilities, enabling control of external devices, such as a robotic ball and a P300-based spelling board, using real-time EEG signals [54].

#### Intervention Effect Evaluation

This category included studies that assessed the effectiveness of educational or therapeutic interventions, with wireless EEG serving as the primary tool for measuring intervention-related neural outcomes. These studies predominantly targeted children with ADHD (n=5), followed by ASD (n=1), ID (n=1), CP (n=1), and OCD (n=1; Figure 3).

In ADHD, noninvasive brain stimulation, including transcranial direct current stimulation and transcranial random noise stimulation, was evaluated using EEG-derived measures [57,58], as were cognitive outcomes associated with a virtual reality–based intervention [73]. In ASD, EEG was applied to examine the effects of music-based interventions on facial emotion recognition [56], whereas in ID, it was used to evaluate attentional outcomes following a structured educational training program [36]. Additionally, Matamoros et al [45] assessed the neural effects of dolphin-assisted therapy in children with CP and OCD.

#### Task or State Monitoring

In total, 5 studies categorized under this domain used wireless EEG to monitor real-time cognitive, affective, and mental states, such as attention, engagement, and mental workload, during naturalistic tasks, therapies, or learning activities, targeting children with ASD (n=2), ID (n=2), and ADHD (n=1; Figure 3).

In the ASD group, mobile EEG successfully tracked dynamic fluctuations in social engagement during therapy [37] and affective states during a virtual reality driving task [79]. In the ID group, real-time state monitoring was used to adjust ambient sensory stimuli based on relaxation and attention levels in an adaptive smart room [77] and to assess brain activity during concentration and resting states in an educational training program [34]. In the ADHD group, wireless EEG was used to evaluate attention and frustration levels during a game-based learning task [38].

### Results of Individual Sources of Evidence II: Wireless EEG Device Characteristics

In the 65 study-device pairs reviewed, 27 distinct wireless EEG devices were identified; detailed specifications are provided in Multimedia Appendix 3. Table 2 presents the technical profiles of wireless EEG devices across 65 study-device pairs (one study [54] used 2 devices). Consumer-grade devices accounted for nearly half of all pairs (31/65, 47.7%), followed by RUO (19/65, 29.2%) and medical devices (15/65, 23.1%). The overall median channel count was 8 (IQR 2-14), with over a third (23/65, 35.4%) using 2 or fewer channels. Dry electrodes were the most common type (26/65, 40%), and headsets were the predominant device design (38/65, 58.5%). The median sampling rate was 500 Hz (IQR 250-512), though 18.5% (12/65) of pairs did not report this parameter.

**Table 2.** Technical profiles of wireless EEG^a^ devices stratified by application purpose across 65 study-device pairs (one study [54] used 2 devices)^b^.

Characteristic	Biomarker-driven assessment (n=33)	BCI^c^ technology (n=19)	Intervention evaluation (n=8)	Task or state monitoring (n=5)	Total (n=65)
Unique devices, n	15	12	5	4	27
Most frequently used devices, n (%)					
First	9 (27.3)^d^	3 (15.8)^e^	3 (37.5)^f^	2 (40)^g^	13 (20)^g^
Second	7 (21.2)^g^	2 (10.5)^h^	2 (25)^g^	1 each (20)^i^	9 (13.8)^d^
Regulatory status, n (%)					
Consumer-grade	13 (39.4)	11 (57.9)	4 (50)	3 (60)	31 (47.7)
Medical device	7 (21.2)	4 (21.1)	3 (37.5)	1 (20)	15 (23.1)
Research use only	13 (39.4)	4 (21.1)	1 (12.5)	1 (20)	19 (29.2)
Channel count					
Median (IQR)	8 (7‐14)	2 (2‐4)	11 (2‐19)	14 (2‐14)	8 (2‐14)
≤2, n (%)	4 (12.1)	14 (73.7)	3 (37.5)	2 (40)	23 (35.4)
3‐14, n (%)	24 (72.7)	5 (26.3)	3 (37.5)	2 (40)	34 (52.3)
≥15, n (%)	4 (12.1)	0 (0)	2 (25)	1 (20)	7 (10.8)
Electrode type, n (%)					
Dry	9 (27.3)	12 (63.2)	3 (37.5)	2 (40)	26 (40)
Semidry (saline)	18 (54.5)	2 (10.5)	2 (25)	2 (40)	24 (36.9)
Gel	4 (12.1)	3 (15.8)	2 (25)	0 (0)	9 (13.8)
CoME-D^j^ score					
Median (IQR)	3.0 (3-4)	4.0 (4-4)	3.5 (3-4)	4.0 (3-4)	3.0 (3-4)
Sampling rate, Hz					
Median (IQR)	512 (439‐1000)	250 (160‐512)	325 (220‐503)	450 (332‐503)	500 (250‐512)
Reported, n (%)	28 (84.8)	13 (68.4)	8 (100)	4 (80)	53 (81.5)
Device design, n (%)					
Headband	1 (3)	8 (42.1)	0 (0)	0 (0)	9 (13.8)
Headset	23 (69.7)	8 (42.1)	4 (50)	3 (60)	38 (58.5)
Cap	6 (18.2)	1 (5.3)	3 (37.5)	1 (20)	11 (16.9)

a EEG: electroencephalography.

b Percentages may not sum to 100% due to unreported values.

c BCI: brain-computer interface.

d BR8 was used.

e Mindwave was used. Four BCI studies used an unspecified research-use-only device (1‐2 channels) and were excluded from device ranking. Among identifiable devices, Mindwave (Neurosky) ranked first.

f g.Nautilus was used.

g EPOC (Emotiv EPOC series [EPOC, EPOC+, and EPOC-X]) was used.

h OmniCNS was used.

i Three devices were tied in the task or state monitoring category: Custom Wireless EEG, Enobio (Neuroelectrics), and Mindwave.

j CoME-D: Categorization of Mobile EEG Devices; a device mobility score ranging from 0=off-body to 5=head-mounted with no additional equipment.

When stratified by application purpose, distinct device selection patterns emerged. BCI studies predominantly used low-channel (median 2, IQR 2‐4), dry electrodes (12/19, 63.2%), and consumer-grade devices (11/19, 57.9%) in headband style (8/19, 42%)—a form factor limited to frontal cortical coverage. These choices reflect a prioritization of ease of use and rapid deployment over spatial resolution, as further indicated by the highest mobility scores across all categories (CoME-D median 4.0, IQR 4‐4). In contrast, biomarker-driven studies required higher channel counts (median 8, IQR 7‐14), higher sampling rates (median 512 Hz, IQR 439‐1000), and semidry electrodes (18/33, 54.5%), predominantly in headset style (23/33, 69.7%). Consumer-grade and RUO devices were equally represented (13/33, 39.4% each); notably, 69.2% (9/13) of RUO pairs were attributed to the BR8 system used by a single research group across 9 studies, warranting caution in generalizing this category’s technical profile. Intervention evaluation studies were characterized by higher channel counts (median 11, IQR 2‐19) alongside the largest proportion of medical devices (3/8, 37.5%) and cap designs (3/8, 37.5%)—a full-scalp configuration enabling broader spatial resolution—suggesting a preference for higher signal fidelity when objectively measuring treatment effects. Notably, both BCI technology and task or state monitoring studies had the highest median CoME-D scores (median 4.0, IQR 4-4; median 4.0, IQR 3-4, respectively), reflecting the shared requirement for maximal device mobility.

### Synthesis of Results I: Neurobehavioral Challenges to Device and Methodological Approaches

Table 3 maps the neurobehavioral challenges associated with EEG recording in children with DDs to the corresponding device and methodological considerations reported in the reviewed literature.

**Table 3.** Suitability of wireless EEG^a^ devices and methodological approaches for addressing specific neurobehavioral challenges during EEG recording in children with developmental disabilities.

Clinical feature	Target population	Challenges during EEG recording	Device suitability and methodological approaches
Learning and behavior			
Inattention and hyperactivity	ADHD^b^, ASD^c^	Lengthy traditional setup with gel electrode exhausts the child’s limited attention span before data acquisition begins. High risk of motion artifacts from fidgeting.	Rapid setup: Wireless devices with dry or semidry sponge sensors enable 1‐ to 5-minute setups, bypassing tedious skin preparation [37,46]. Artifact mitigation: Advanced EEG artifact removal methods, such as ICA^d^ and ASR^e^, can reduce motion-related artifacts and improve the isolation of neural signals [46,52,65,67,88,96].
Cognitive processing difficulties	ADHD, ID^f^, LD^g^	High susceptibility to sensory and cognitive overload, leading to anxiety during testing. Difficulty comprehending complex instructions and maintaining attention during cognitive evaluation.	Adaptive “smart space” paradigms: Environments that dynamically adjust ambient stimuli (eg, lighting and music) based on real-time EEG-derived attention metrics to mitigate overload [77]. Nonthreatening form factor: Child-friendly devices such as headphone-like designs or helmets with the appearance of an animal or a cartoon may reduce test-related anxiety and visual overload by resembling consumer electronics rather than medical equipment [46,77]. Integrated mobile app platform: Combining single-channel wireless EEG (via Bluetooth) with a child-friendly digitized cognitive test in a single mobile app can increase willingness to participate and simplify procedural demands [44].
Sensory hypersensitivity	ASD, CP^h^	Tactile discomfort and distress triggered by conductive gel, tight caps, or abrasive skin preparation. High emotional arousal and behavioral outbursts in overstimulating settings.	Sensory-friendly hardware: Soft fabric caps and dry electrodes can minimize tactile defensiveness [53]. Desensitization protocols: Introducing the cap as a “swimming hat” and using stepwise exposure to gel when gel-based electrodes are required [53,62].
Social			
Social interaction impairments	ASD	Difficulty interacting with unfamiliar clinicians or researchers, which can trigger severe social anxiety and hinder task performance.	Familiar mediators and play-based paradigms: Using familiar mediators (parents or therapists) and seminaturalistic play or AR^i^-based paradigms instead of rigid laboratory protocols [61]. Home-based monitoring: Transitioning to naturalistic environments may reduce emotional arousal and capture authentic neural activity [53,62,94].
Motor			
Involuntary movements and postural constraints	CP, severe motor disorders	Difficulty maintaining stable electrode placement due to involuntary spasms. Physical stabilization by caregivers can introduce mechanical noise. Wheelchair headrests or supportive positioning devices may interfere with headset placement.	Flexible and ergonomic support: Wireless headsets allow the use of ergonomic physical support (eg, beanbag chairs) that naturally support the head or neck without the restriction of a wired tether, which minimizes unwanted noise [54]. Adaptation of surrounding equipment: Removing or modifying interfering wheelchair headrests may improve headset fit and recording stability [54].

a EEG: electroencephalography.

b ADHD: attention-deficit/hyperactivity disorder.

c ASD: autism spectrum disorder.

d ICA: independent component analysis (a computational method for separating multichannel signals into additive subcomponents).

e ASR: artifact subspace reconstruction (an automated method to remove nonstationary high-amplitude artifacts).

f ID: intellectual disability.

g LD: learning disorder.

h CP: cerebral palsy.

i AR: augmented reality.

Inattention and hyperactivity, commonly observed in children with ADHD and often co-occurring in those with ASD, pose challenges, including limited attentional capacity exhausted by lengthy conventional setup procedures and high motion artifact risk from fidgeting. Rapid-setup wireless devices with dry or semidry sensors have been adopted to reduce the preparation time [37,46]. However, as dry electrodes are generally more susceptible to motion-related signal contamination, several studies applied artifact rejection techniques, such as ICA and ASR, to improve signal quality [46,52,65,67,88,96].

Cognitive processing difficulties, particularly for children with ADHD, ID, and LD, who may be susceptible to sensory and cognitive overload, presented further challenges during EEG acquisition. Three strategies have been proposed to address these concerns. First, adaptive “smart space” paradigms—environments that dynamically adjust ambient stimuli (eg, lighting and music) based on real-time EEG-derived attention metrics—have been suggested to mitigate cognitive overload [77]. Second, child-friendly device features may help reduce test anxiety and visual overload [46,77]. For example, EEG devices designed to resemble headphone-like headsets or animal- or cartoon-themed helmets, rather than conventional medical equipment, may be more acceptable to children [77]. Third, children with ID or LD may have difficulty comprehending complex instructions. To address this, a mobile app that integrates wireless EEG with a child-friendly digital cognitive test has been proposed as a strategy to increase children’s willingness to participate [44].

Sensory hypersensitivity, often reported in children with ASD, manifests as tactile discomfort and distress triggered by conductive gel and tight-fitting caps. To minimize tactile defensiveness, some studies adopted sensory-friendly hardware, including soft fabric caps and dry electrodes [53]. In cases requiring gel-based electrodes to ensure adequate signal quality, behavioral desensitization strategies were explored as a complementary approach, such as introducing the cap as a “swimming hat” and applying stepwise gel exposure [53,62].

Social interaction impairments, predominantly in children with ASD, included difficulties engaging with unfamiliar researchers, which may interfere with task compliance. To address this, several studies involved familiar caregivers or mediators and adopted play-based or augmented reality paradigms to reduce social demands [61], whereas others transitioned to home-based monitoring environments to increase ecological comfort [53,62,94].

Challenges in maintaining stable electrode placement include involuntary movements and postural constraints, characteristics of children with CP, and severe motor disorders. Flexible wireless headsets combined with ergonomic supports (eg, beanbag chairs) may help stabilize electrode positioning and reduce mechanical noise from involuntary movements [54]. Removing or modifying surrounding supportive equipment, such as wheelchair headrests or positioning devices, may further improve headset fit and recording stability [54].

### Synthesis of Results II: Data Quality–Related Reporting Characteristics

**Figure 4.**
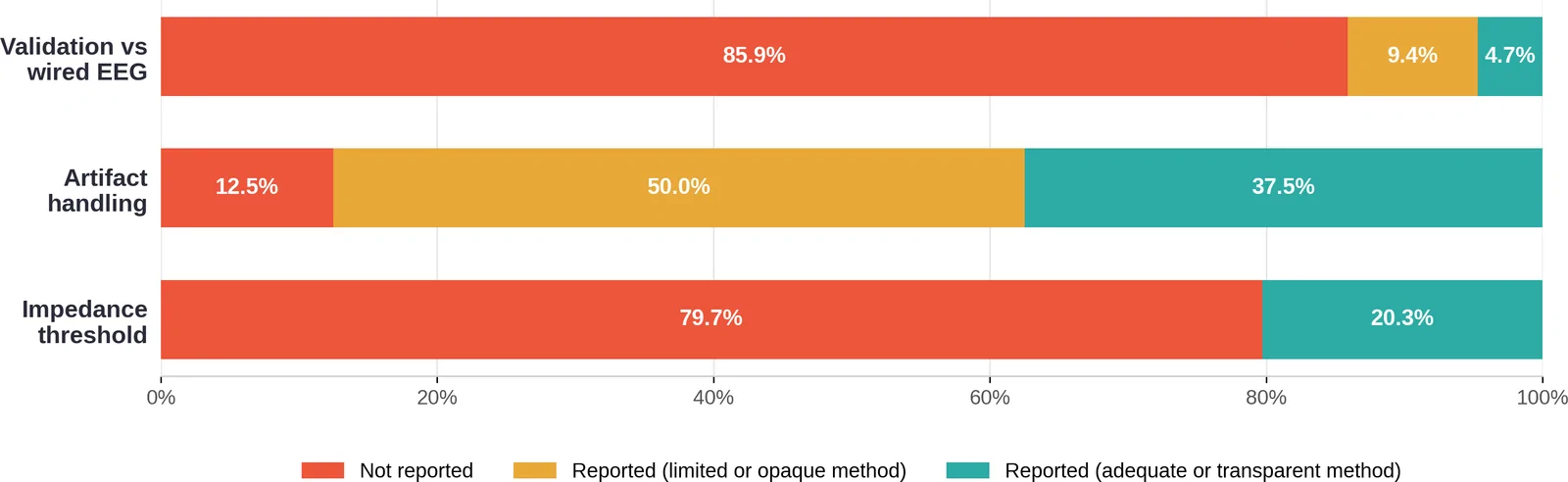
Data quality–related reporting characteristics of the reviewed studies (N=64). Stacked bars represent the proportion of studies classified by reporting completeness for 3 domains: validation against a gold-standard wired EEG system, artifact handling methods, and impedance threshold specification. Not reported (red) indicates no information was provided; reported with limited or opaque method (yellow) indicates reliance on indirect validation or proprietary or nontransparent artifact processing; reported with adequate or transparent method (teal) indicates direct validation, use of established signal processing techniques (eg, independent component analysis and artifact subspace reconstruction), or specification of explicit impedance values. EEG: electroencephalography.

Of the 64 reviewed studies, data quality–related reporting was substantially incomplete across all 3 domains examined (Figure 4). Full extraction details for each study, including a summary of data quality–related reporting characteristics, are provided in Multimedia Appendix 4.

Validation against a gold-standard wired EEG system was the most poorly reported (Figure 4). Most studies (55/64, 85.9%) did not report any validation. In total, 6 (9.4%) studies relied on indirect validation, citing prior technical studies predominantly conducted in neurotypical adults to support device reliability. Only 3 (4.7%) studies conducted direct validation against a gold-standard system. Specifically, Bach-Morrow et al [49] conducted head-to-head comparisons with conventional wired EEG.

Artifact handling methods were reported in the majority of studies (56/64, 87.5%); however, the transparency of the methods used varied considerably (Figure 4). In total, 19 (29.7%) studies used established blind source separation techniques such as ICA or ASR. An additional 5 (7.8%) studies used other established signal processing approaches, including wavelet transform, iterative template matching and suppression, and source imaging–based methods. In contrast, an equal number of studies (19/64, 29.7%) relied on proprietary algorithms embedded in consumer-grade devices, and 13 (20.3%) studies used only basic filtering or manual segment rejection—both of which offer limited methodological transparency. The remaining 8 (12.5%) studies did not report any artifact handling procedure.

Impedance thresholds, defined as the maximum acceptable electrode-scalp resistance specified to ensure adequate signal quality during EEG recording, were also poorly reported. Many studies (51/64, 79.7%) did not specify any impedance threshold (Figure 4). Among the studies that reported impedance, the most common threshold was <100 kΩ (10/64, 15.6%), followed by <30 kΩ (2/64, 3.1%) and <20 kΩ (1/64, 1.6%).

## Discussion

### Summary of Evidence

To the best of our knowledge, this is the first study to comprehensively map the use of wireless EEG technology in children across a broad spectrum of DDs, rather than limiting the scope to a single DD. This broader scope highlights the growing interest in wireless EEG application in this population, as their use is not constrained by the study setting or physical environment, which reflects a broader global trend toward the use of wireless and mobile EEG technology [19,20].

In this study, key trends in the primary applications of wireless EEG devices were identified: biomarker-driven clinical assessment and diagnosis, BCI technology, intervention effect evaluation, and task or state monitoring. Wireless EEG was predominantly used to identify neurophysiological biomarkers associated with DDs and support clinical diagnosis, particularly in ADHD and ASD, whose core symptoms are fundamentally tied to environmental triggers and social contexts. The tethering effect of conventional wired EEG may constrain the ecological validity of neural recordings by limiting natural behavior.

However, available evidence in the present review focuses on ADHD and ASD, with limited research on other DDs, such as global developmental delay, tics or Tourette syndrome, or LD. This imbalance likely reflects a broader pattern in the existing literature rather than a limitation specific to this review. Previous systematic reviews have similarly reported that technology-based and neurophysiological research in neurodevelopmental disorders is disproportionately concentrated on ASD and ADHD [99]. Moreover, the reviewed studies very rarely include comparative designs [100], limiting the ability to determine whether observed EEG features are disorder-specific or reflect broader neurodevelopmental characteristics. Recent studies beyond the scope of this review have identified EEG as a promising tool for biomarker assessment in other DD populations, including ERP assessment in Tourette syndrome [101] and objective evaluation of disease severity in rare genetic disorders such as Rett syndrome and fragile X syndrome [102]. Future studies should further investigate neurophysiological biomarkers across a broader range of DDs to improve the disease and symptom specificity of wireless EEG-based assessments.

Most studies targeted school-aged children, with limited inclusion of toddlers and preschoolers. Only 2 studies evaluated early ASD screening before age 3 years using combined psychological data and physiological signals recorded with wireless EEG [55,72]. This gap is critical, as early detection of DDs enables timely intervention by leveraging the heightened neuroplasticity of the first 3 years of life [103,104]. However, EEG research with children younger than 3 years of age remains scarce, largely due to the high attrition rates associated with traditional wired systems, driven by cap refusal and movement artifacts [105]. These barriers are further compounded in DD populations, particularly in ASD, in which sensory hypersensitivity reduces tolerance for conventional equipment (Table 3). Wireless EEG, which features sensory-friendly hardware, such as fabric caps or dry electrodes, may help mitigate these barriers [15-17,53].

Regarding the EEG recording environment, although approximately two-thirds of the included studies were conducted in clinical settings, likely reflecting hospital-based recruitment of children with DDs, this review identified a growing shift toward home-based EEG studies. These studies aimed to enable sleep monitoring [62] and capture neurophysiological activity in real-life contexts [53]. Given the cognitive, sensory, and motor sensitivities of children with DDs, the use of wireless EEG technology in familiar environments may provide an effective strategy for improving participant compliance [16]. This trend extends to neurofeedback training, in which home-based delivery via wireless EEG on tablet or mobile platforms has been proposed as a means to bridge the treatment gap and enhance accessibility without requiring high-cost on-site therapist supervision [106].

Furthermore, most studies were conducted in Asian and Western countries, consistent with previous findings [20], while no studies were identified from Africa, a region accounting for over half of all DD cases globally [1]. However, these regions face a chronic shortage of neurophysiological researchers and diagnostic infrastructure [107]. Although emerging evidence suggests that deployment of cost-effective wireless EEG systems is feasible in low-resource settings [105,108], these studies have targeted typically developing children, and the applicability of wireless EEG for DD populations in resource-limited contexts remains unexamined. Addressing this gap is essential to improve the global generalizability of wireless EEG research in children with DDs.

The predominance of consumer-grade wireless EEG devices, such as Emotiv EPOC and NeuroSky MindWave, across the reviewed studies, consistent with previous findings [19,20,109], likely reflects their lower initial hardware investment and commercial accessibility rather than superior signal quality. However, the cross-tabulation revealed that this reliance on a small number of devices is not uniform across research contexts; rather, device selection is systematically aligned with research purpose. BCI studies consistently favored participant comfort and rapid deployment over spatial resolution, whereas biomarker-driven diagnostic studies and intervention evaluation studies both prioritized signal fidelity—the former to capture subtle neurophysiological signatures, the latter to ensure robust outcome measurement when assessing treatment effects. This purpose-driven divergence is consistent with the broader wireless EEG literature: Niso et al [19] observed that device selection is shaped by a trade-off between ecological validity and signal fidelity, and Sabio et al [20] reported that consumer-grade devices are predominantly used for BCI and experimental research rather than clinical applications.

Importantly, even within the consumer-grade category, performance is not equivalent; Maskeliunas et al [110] demonstrated that the EPOC achieved higher recognition accuracy than the MindWave for attention and eye-blink detection tasks, underscoring that these devices are not interchangeable. Furthermore, consumer-grade systems are not cleared as medical devices and may not meet the technical standards required for clinical decision-making [109,111], and the proprietary nature of some systems may limit methodological transparency and third-party data access [109]. That said, consumer-grade devices have demonstrated utility in neurofeedback training paradigms targeting attentional outcomes [106], where the primary goal is to discriminate relative cognitive states in real time rather than to achieve diagnostic-level signal fidelity.

Nevertheless, evidence on device suitability and recording barriers was uneven across diagnostic groups; studies involving ID, CP, and LD were few and generally relied on 1- or 2-channel consumer-grade devices [39,45,54,77], meaning that the limited reporting of EEG recording difficulties in these populations should not be interpreted as evidence of fewer challenges. Future research would benefit from explicit reporting of the rationale for device selection and the contextual adaptations used, enabling subsequent reviews to distinguish preference-driven from evidence-driven choices.

A central contribution of this review is the systematic assessment of data quality–related reporting across the reviewed studies, which revealed pervasive and consequential gaps. Only 3 (4.7%) studies reported direct validation of their wireless EEG device against a medical-grade wired system within the target pediatric DD cohort, and only about 1 in 5 (13/64, 20.3%) specified impedance thresholds during recording. Critically, existing validation has been largely derived from adults or typically developing children [112,113], and its applicability to DD populations requires cautious interpretation. Even within neurotypical samples, validation studies have yielded inconsistent results [16,111,113]. Compounding these concerns, no consensus has been established on benchmarking criteria for evaluating wireless EEG systems, making cross-study comparisons challenging [16]. Beyond comparability, the reproducibility of reported findings is equally at risk: Troller-Renfree et al [114] documented substantial heterogeneity in analytic pipelines across pediatric EEG studies—from preprocessing parameters to artifact rejection criteria. Our review extends these observations to the wireless EEG context, where the combination of lower-density electrode arrays, dry or semidry sensor technologies, and behaviorally challenging pediatric populations introduces additional sources of signal variability that are rarely characterized or reported. Taken together, without transparent reporting, the reliability of wireless EEG findings in this population cannot be adequately assessed [115,116].

### Limitations

This review has several limitations. First, it was restricted to full-text, English-language publications and excluded gray literature, unpublished or ongoing studies, guidelines, protocols, and qualitative studies. These restrictions may have led to the omission of relevant informal data and increased the risk of publication bias. Second, the included studies were predominantly conducted in Asian and Western countries, with Taiwan accounting for 23.4% (15/64) of the studies. This concentration of research output from a limited number of research groups may have introduced geographic bias. Third, consistent with scoping review methodology, a formal quality appraisal of individual studies was not conducted; therefore, the findings should be interpreted in light of varying methodological rigor across the included studies. Fourth, while the lower initial acquisition cost of consumer-grade wireless devices was frequently cited as a practical advantage in the included studies, none conducted a formal cost-effectiveness analysis or assessed the total cost of ownership relative to conventional wired systems. Dedicated cost-effectiveness analyses comparing wireless and wired EEG systems in pediatric DD research are needed. Finally, the lack of direct comparative validation against gold-standard systems within the target cohorts remains a critical gap.

Despite these limitations, the primary value of this review lies in its broad synthesis of wireless EEG research in children with DDs, identification of data quality–related reporting gaps, and proposal of directions for future research.

### Conclusions

This scoping review mapped the current research landscape and provided a structured overview of wireless EEG applications in children with DDs. The reviewed studies suggest that wireless EEG has been applied in research on biomarker-driven clinical assessment and diagnosis, BCI applications, intervention evaluation, and task or state monitoring, particularly in ADHD and ASD. However, this review identified substantial gaps in data quality–related reporting, such as the underreporting of impedance thresholds and reliance on nontransparent artifact handling methods, which limit the interpretability and comparability of current findings. The technical limitations of wireless EEG, such as lower signal‐to‐noise ratio and susceptibility to motion artifacts, remain important considerations. Future studies should prioritize rigorous validation within DD cohorts and the development of population-specific guidelines for device selection, signal quality assurance, and reporting transparency.

## Supplementary material

10.2196/84707 Multimedia Appendix 1 Search strategies by category.

10.2196/84707 Multimedia Appendix 2 Summary of the main characteristics of the included studies.

10.2196/84707 Multimedia Appendix 3 Technical specifications of all wireless electroencephalography devices identified across 65 study–device pairs in the reviewed studies.

10.2196/84707 Multimedia Appendix 4 Data quality–related reporting characteristics of included studies on wireless electroencephalography in children with developmental disabilities.

10.2196/84707 Checklist 1 PRISMA-ScR checklist.1

## Abbreviations

ADHD: attention-deficit/hyperactivity disorder
ASD: autism spectrum disorder
ASR: artifact subspace reconstruction
BCI: brain-computer interface
CoME-D: Categorization of Mobile EEG Devices
CP: cerebral palsy
DD: developmental disability
EEG: electroencephalography
ERP: event-related potential
ICA: independent component analysis
ID: intellectual disability
LD: learning disorder
OCD: obsessive-compulsive disorder
PRISMA-S: PRISMA Statement for Reporting Literature Searches in Systematic Reviews
PRISMA-ScR: Preferred Reporting Items for Systematic Reviews and Meta-Analyses extension for Scoping Reviews
RUO: research-use-only
