# Supplementary material for: Wireless Electroencephalography in Research on Children With Developmental Disabilities: Scoping Review
Source: J Med Internet Res. 2026 Aug 25;28:e84707. doi: 10.2196/84707 (PMC13505603; doi:10.2196/84707)
Supplement: Supplementary file 1 [file jmir-v28-e84707-s001.docx]

**Search Strategies by Category.**

|  | Category 1 | Category 2 | Category3 |
| --- | --- | --- | --- |
| Title | **EEG** | **Child** | **DD** |
| PubMed | (   ( ( ambulatory[Title/Abstract] OR mobile[Title/Abstract] OR portable[Title/Abstract] OR wearable[Title/Abstract] OR wireless[Title/Abstract] OR "consumer-grade"[Title/Abstract]) AND (electroencephalog*[Title/Abstract] OR EEG[Title/Abstract]) )  OR  ( ( EEG[Title/Abstract] OR electroencephalog*[Title/Abstract] ) AND ( Muse[All Fields] OR Emotiv[All Fields] OR NeuroSky[All Fields] OR OpenBCI[All Fields] OR Neuroelectrics[All Fields] OR Dreem[All Fields] OR "ANT Neuro"[All Fields] OR Bitbrain[All Fields] OR Compumedics[All Fields] OR "g.tec"[All Fields] OR Mitsar ) )   ) | ( "child"[MeSH Terms] OR "adolescent"[MeSH Terms] OR "infant"[MeSH Terms] OR "child*"[Title/Abstract] OR "pediatric"[Title/Abstract] OR "toddler"[Title/Abstract] OR "preschool"[Title/Abstract] OR "adolescent"[Title/Abstract] OR "teenager"[Title/Abstract] OR "youth"[Title/Abstract] OR "infant"[Title/Abstract] ) | ("developmental disabilities"[MeSH Terms] OR "developmental dis*"[Title/Abstract] OR "intellectual disability"[MeSH Terms] OR "intellectual dis*"[Title/Abstract] OR "autism spectrum disorder"[MeSH Terms] OR "ASD"[Title/Abstract] OR "attention deficit disorder with hyperactivity"[MeSH Terms] OR "ADHD"[Title/Abstract] OR "down syndrome"[MeSH Terms] OR "down syndrome"[Title/Abstract] OR "cerebral palsy"[MeSH Terms] OR "cerebral palsy"[Title/Abstract] OR "learning dis*"[Title/Abstract]) |
| Web of Science | (   ( TS=(EEG OR electroencephalog*) AND TS=(ambulatory OR mobile OR portable OR wearable OR wireless OR "consumer-grade") )   OR   ( TS=(EEG OR electroencephalog*) AND ALL=(Muse OR Emotiv OR NeuroSky OR OpenBCI OR Neuroelectrics OR Dreem OR "ANT Neuro" OR Bitbrain OR Compumedics OR "g.tec" OR Mitsar) )   ) | TS=( child* OR pediatric OR toddler OR preschool OR adolescent OR teenager OR youth OR infant ) | TS=( "developmental dis*" OR "intellectual dis*" OR "autism spectrum disorder" OR ASD OR "attention deficit disorder with hyperactivity" OR ADHD OR "down syndrome" OR "cerebral palsy" OR "learning dis*") |
| EMBASE | (     ( ( electroencephalog*:ti,ab,kw OR EEG:ti,ab,kw) AND ( ambulatory:ti,ab,kw OR mobile:ti,ab,kw OR portable:ti,ab,kw OR wearable:ti,ab,kw OR wireless:ti,ab,kw OR 'consumer-grade':ti,ab,kw ) )     OR     ( ( EEG:ti,ab,kw OR electroencephalog*:ti,ab,kw ) AND ( Muse OR Emotiv OR NeuroSky OR OpenBCI OR Neuroelectrics OR Dreem OR ‘ANT Neuro’ OR Bitbrain OR Compumedics OR ‘g.tec’ OR Mitsar ) )  ) | ( 'child*':ti,ab,kw OR 'pediatric':ti,ab,kw OR 'toddler':ti,ab,kw OR 'preschool':ti,ab,kw OR 'adolescent':ti,ab,kw OR teenager:ti,ab,kw OR 'youth':ti,ab,kw OR 'infant':ti,ab,kw ) | ( 'developmental dis*':ti,ab,kw OR 'intellectual dis*':ti,ab,kw OR 'autism spectrum disorder':ti,ab,kw OR ASD:ti,ab,kw  OR 'attention deficit disorder with hyperactivity':ti,ab,kw OR ADHD:ti,ab,kw OR 'down syndrome':ti,ab,kw OR 'cerebral palsy':ti,ab,kw OR 'learning dis*':ti,ab,kw ) |
| CINAHL | (  ( (TI ambulatory OR TI mobile OR TI portable OR TI wearable OR TI wireless OR TI "consumer-grade"   OR AB ambulatory OR AB mobile OR AB portable OR AB wearable OR AB wireless OR AB "consumer-grade") AND (TI EEG OR TI electroencephalog* OR AB EEG OR AB electroencephalog*) )  OR   ( (TI EEG OR TI electroencephalog* OR AB EEG OR AB electroencephalog*) AND (Muse OR Emotiv OR NeuroSky OR OpenBCI OR Neuroelectrics OR Dreem OR "ANT Neuro" OR Bitbrain OR Compumedics OR "g.tec" OR Mitsar) )  ) | (MH child OR TI child* OR TI pediatric OR TI toddler OR TI preschool OR TI adolescent OR TI youth OR TI infant OR AB child* OR AB pediatric OR AB toddler OR AB preschool OR AB adolescent OR AB teenager OR AB youth OR AB infant) | (MH “developmental disabilities” OR TI "developmental dis*" OR TI "intellectual dis*" OR TI "autism spectrum disorder" OR TI ASD OR TI "attention deficit disorder with hyperactivity" OR TI ADHD OR TI "down syndrome" OR TI "cerebral palsy" OR TI "learning dis*" OR AB "developmental dis*" OR AB "intellectual dis*" OR AB "autism spectrum disorder" OR AB ASD OR AB "attention deficit disorder with hyperactivity" OR AB ADHD OR AB "down syndrome" OR AB "cerebral palsy" OR AB "learning dis*") |
| PsycInfo | (     ( ( tiab(electroencephalog*) OR tiab(EEG) ) AND ( tiab(ambulatory) OR tiab(mobile) OR tiab(portable) OR tiab(wearable) OR tiab(wireless) OR tiab("consumer-grade") ) )     OR     ( ( tiab(EEG) OR tiab(electroencephalog*)) AND (Muse OR Emotiv OR NeuroSky OR OpenBCI OR Neuroelectrics OR Dreem       OR "ANT Neuro" OR Bitbrain OR Compumedics OR "g.tec" OR Mitsar) )  ) | (tiab(child*) OR tiab(pediatric) OR tiab(toddler) OR tiab(preschool) OR tiab(adolescent) OR tiab(teenager) OR tiab(youth) OR tiab(infant)) | ( tiab("developmental dis*") OR tiab("intellectual dis*") OR tiab("autism spectrum disorder") OR tiab(ASD) OR tiab("attention deficit disorder with hyperactivity") OR tiab(ADHD) OR tiab("down syndrome") OR tiab("cerebral palsy") OR tiab("learning dis*") ) |
| IEEExplore | (     ( (ambulatory OR mobile OR portable OR wearable OR wireless OR "consumer-grade") AND (electroencephalography OR electroencephalogram OR EEG) )  OR     ( (electroencephalography OR electroencephalogram OR EEG) AND (Muse OR Emotiv OR NeuroSky OR OpenBCI OR Neuroelectrics OR Dreem OR "ANT Neuro" OR Bitbrain OR Compumedics OR "g.tec" OR Mitsar) )  ) | (child OR pediatric OR toddler OR preschool OR adolescent OR teenager OR youth OR infant) | ("developmental dis*" OR "intellectual dis*" OR "autism spectrum disorder" OR ASD OR "attention deficit disorder with hyperactivity" OR ADHD OR "down syndrome" OR "cerebral palsy" OR "learning dis*") |
| Scopus | (     ( TITLE-ABS-KEY(electroencephalog* OR EEG)       AND TITLE-ABS-KEY(ambulatory OR mobile OR portable OR wearable OR wireless OR "consumer-grade") )     OR     ( TITLE-ABS-KEY(EEG OR electroencephalog*)       AND TITLE-ABS-KEY(Muse OR Emotiv OR NeuroSky OR OpenBCI OR Neuroelectrics OR Dreem OR "ANT Neuro" OR Bitbrain OR Compumedics OR "g.tec" OR Mitsar) )  ) | TITLE-ABS-KEY(child* OR pediatric OR toddler OR preschool OR adolescent OR teenager OR youth OR infant) | TITLE-ABS-KEY("developmental dis*" OR "intellectual dis*" OR "autism spectrum disorder" OR ASD   OR "attention deficit disorder with hyperactivity" OR ADHD OR "down syndrome" OR "cerebral palsy"   OR "learning dis*") |
