# Supplementary material for: Wireless Electroencephalography in Research on Children With Developmental Disabilities: Scoping Review
Source: J Med Internet Res. 2026 Aug 25;28:e84707. doi: 10.2196/84707 (PMC13505603; doi:10.2196/84707)
Supplement: Supplementary file 2 [file jmir-v28-e84707-s002.docx]

| Study [reference number] | Year | Country (where the study was conducted) | EEG recording environment | Study design | Study objective | EEG data analysis | Main finding | Population | Age |
| --- | --- | --- | --- | --- | --- | --- | --- | --- | --- |
| Modarres-Zadeh et al [33] | 2005 | USA | Clinic or lab | Observational study (cross-sectional & pre-post) | To develop a neuro-behavioral test for the quantitative evaluation of ADHD and for gauging the effectiveness of medication using neurobehavioral indices. | Time-frequency analysis (STFT), Frequency analysis (theta/alpha ratio) | The combined neuro-behavioral index was significantly more discriminative (normal vs. pre-medication ADHD) and more sensitive to medication than the indices based solely on EEG or behavioral responses. | 5 ADHD, 5TD | 10-17 |
| Iramina et al [34] | 2010 | Japan | Clinic or lab | Observational study (case study) | To investigate brain activity in concentration and resting states during an educational program. | Time-frequency analysis (STFT & wavelet transform) | There were differences in EEG power in the alpha and theta bands between the concentration and resting states. | 1 ID | 10 |
| Lim et al [75] | 2012 | Singapore | Clinic or lab | Experimental study (non-randomized controlled trial) | To investigate the effectiveness and acceptability of an enhanced BCI–based attention training system that combines dry EEG sensors with a novel game. | Time-frequency Analysis (Fast-Time-Fourier Transform) | A BCI-based attention training program significantly improved clinical symptoms in children with ADHD who were not taking medication, and these effects were maintained over the long term. | 20 ADHD | 6-12 |
| Israsena et al [35] | 2015 | Thailand | Educational institution | Experimental study (non-randomized controlled trial) | To evaluate an EEG-based neurofeedback game (BrainUp) designed to train attention-related cognitive tasks. | Frequency analysis (Beta/Alpha ratio) | BrainUp significantly improved attention in both groups, with increased beta activity and decreased alpha activity. | 8 ADHD, 20 TD | 7 |
| Jiang and Johnstone [76] | 2015 | China | home | Experimental study (case study) | To evaluate the feasibility and outcomes of combined cognitive and neurofeedback training on the behavior of children with ADHD in China. | Proprietary algorithm (attention level) | The objective and subjective data suggested that neurocognitive training had the potential to improve psychological abilities and behaviors associated with ADHD. | 5 ADHD | 8.1-11.7 |
| Zennifa et al [36] | 2015 | Japan | Clinic or lab | Observational study (longitudinal case study) | To investigate the effects of a structured educational training program on the cognitive state over a period of four years. | Frequency analysis (theta and beta power) | The results in the studying state showed that theta and beta activity increased over four years, demonstrating the effect of educational training on the neurophysiology of children with intellectual disability. | 1 ID | 9-15 |
| Billeci et al [37] | 2016 | Italy | Clinic or lab | Observational study (feasibility pilot) | To distinguish engagement and disengagement states by using EEG and ECG. | Frequency analysis (PSD), connectivity analysis (coherence) | During engagement, EEG power (frontal delta, parietal beta, occipital bands) and coherence (theta, alpha, and beta bands) increased. | 5 ASD | 6-8 |
| Garzotto et al [77] | 2016 | Italy | Clinic or lab | Observational study (methodological / system validation) | To develop and evaluate an EEG-based adaptive smart environment that automatically adjusts therapeutic stimuli based on attention and relaxation levels in children with ID. | Proprietary algorithm (attention level) | The Magic K-Room can automatically adapt stimuli based on EEG-detected relaxation levels, thereby providing objective and quantitative data for therapists. | 20 ID | 7-14 |
| Martínez et al [38] | 2016 | Mexico | Clinic or lab | Observational study (case study) | To understand the affective and cognitive states during educational game-based tasks. | Frequency analysis (alpha, beta, theta, delta) | Engagement and disengagement were distinguished using spectral rhythms. Frustration increased during Leap Motion-based tasks. | 15 ADHD | Not reported |
| Perales et al [39] | 2017 | Spain | Clinic or lab | Observational study (cross-sectional case-control) | To explore which brain waves are involved in the attention process when playing a serious game. | Frequency analysis (band power, TBR) | The CP group showed generally lower EEG power and higher TBR. Soft music produced the lowest TBR, indicating improved attention. | 10 CP, 8 TD | 8-16 |
| Askari et al [40] | 2018 | Iran | Clinic or lab | Observational study (methodological study) | To develop a CNN-based model using EEG energy and entropy features for automatic ASD detection. | Time-frequency analysis (wavelet transform), Complexity analysis (log-entropy) | The model was able to distinguish ASD from TD with an accuracy of 95.1%, based on CNN-derived features classified using a SVM. | 100 ASD, 100 TD | 5-12 |
| Eroğlu et al [41] | 2018 | Türkiye | Clinic or lab | Experimental study (case study) | To evaluate the effect of a multisensory neurofeedback app (AutoTrainBrain) on cognitive function. | Frequency analysis (PSD), connectivity analysis (coherence), complexity analysis (entropy) | AutoTrainBrain improved cognitive function, with increases in relative alpha power, coherence, and entropy. | 1 SLD (Dyslexics) | 14 |
| Fan et al [79] | 2018 | USA | Clinic or lab | Observational study (methodological study) | To build group-level classification models capable of recognizing affective states and mental workload of individuals with ASD during driving skill training. | Time analysis, Frequency analysis (PSD) | Offline EEG-based group-level classification models are feasible for recognizing binary low- and high-intensity affect and workload in individuals with ASD in the context of driving. | 20 ASD | 15.29±1.65 |
| Qian et al [78] | 2018 | Singapore | Clinic or lab | Experimental study (randomized controlled trial) | To elucidate topological alterations in large-scale functional networks induced by 8 weeks of BCI training and to determine their correlation with behavioral alleviation in ADHD children. | Frequency analysis | The training significantly improved inattention and reduced SVN hyperconnectivity. Furthermore, the network topology shifted from a regular toward a random configuration, reflecting a trajectory of facilitated brain maturation. | 29 ADHD (18 intervention, 11 control) | Intervention: 9.00 ± 1.50, Control: 9.45 ± 1.29 |
| Lim et al [81] | 2019 | Singapore | Clinic or lab | Experimental study (randomized feasibility trial) | To assess the impact of an 8-week BCI training (Cogoland) on inattention in unmedicated ADHD subjects compared with a waitlist and to verify the sustained effects over a 12-week maintenance phase. | Time-frequency analysis | The intervention group demonstrated a significant reduction in clinician-rated inattentive symptoms compared with the waitlist group (change: -3.5 vs. -1.9, p = 0.0177). These effects were sustained through week 24 with maintenance training. | 172 ADHD (Intervention: 85, waitlist: 87) | 6-12 |
| Mercado et al [80] | 2019 | Mexico | Clinic or lab | Experimental study (pilot randomized crossover trial) | To develop and pilot-test a BCI video game called "FarmerKeeper". | Proprietary algorithm (attention level) | The training resulted in positive behavioral outcomes, notably decreased anxiety and enhanced attention. | 12 ASD | 4-11 |
| Mercado-Aguirre et al [42] | 2019 | Columbia | Clinic or lab | Observational study (methodological study) | To develop and evaluate a system designed to generate stimuli and to record and analyze cognitive evoked potentials using the Emotiv EPOC+ headset. | Time analysis (ERP) | The system allowed reliable measurement of P300 waves and may provide a more comfortable experience for patients than medical-grade systems. | 12 ADHD, 5 SLD, 2 Both | 8-14 |
| Chu et al [43] | 2020 | Taiwan | Not specified | Observational study (cross-sectional case-control) | To evaluate whether mobile EEG and actigraphy can provide physiological indicators (attention, meditation, activity). | Proprietary algorithm (attention / meditation level) | Although the EEG-based attention index showed significant correlations with CPT performance, actigraphy outperformed EEG in distinguishing children with ADHD from controls. | 49 ADHD, 14 TD | 6-12 |
| Liao et al [44] | 2020 | Taiwan | Not specified | Observational study (cross-sectional case-control) | To assess cognitive performance and brainwave patterns using an EEG-based cognitive testing app. | Frequency analysis | Higher beta and lower delta/theta brainwaves were associated with better cognitive performance. | 11 ADHD/LD, 23 TD | 6-12 |
| Matamoros et al [45] | 2020 | Mexico | Clinic or lab | Experimental study (case study) | To evaluate brain activity before, during, and after DAT. | Frequency analysis (FFT-based PSD), Complexity analysis | DAT induced a significant increase in brain activity. | 3 ISCP, 1 OCD, 1 TD | Not reported |
| Chen et al [46] | 2021 | Taiwan | Clinic or lab | Observational study (cross-sectional case-control) | To identify EEG biomarkers related to attention by analyzing brain dynamics during a task (K-CPT). | Time-frequency analysis (STFT), Frequency analysis (PSD) | The ADHD group showed higher delta power in the central and parietal regions during the slow-rate task, and alpha power decreased from rest to task. | 29 ADHD, 20 TD | 5-7 |
| Johnstone et al [83] | 2021 | China | Clinic and educational institution | Observational study (methodological study) | To assess if the integrated NCAT outperforms single-domain metrics in accurately classifying ADHD-I versus healthy controls. | Frequency analysis | The multimodal model (SNAP-IV, NCAT-core, and EEG) outperformed the other models, with 93.4% accuracy (sensitivity 91.4%, specificity 93.9%). Although EEG alone reached 81.2% accuracy, it proved vital to the combined model, specifically through EO/FO alpha, theta activation, and EO TBR markers. | 53 ADHD, 161 TD | 7-12 |
| Peng et al [47] | 2021 | China | Clinic or lab | Observational study (cross-sectional case-control) | To develop an early screening method for ASD using ERP paradigms under emotional stimuli and feature selection via L1-norm regularized SVM. | Time analysis (ERP), Frequency analysis (spectral power), Complexity analysis | Screening accuracy for ASD was 93.8% under positive emotional stimulation and 87.5% under negative stimulation. The proposed algorithm enhanced this performance by effectively removing redundant EEG features. | 32 ASD, 32 TD | 4-6 |
| Serrano-Barroso et al [48] | 2021 | Spain | Not specified | Observational study (cross-sectional case-control) | To evaluate whether an EEG-based neurofeedback game (GokEvolution) can effectively detect attention levels. | Proprietary algorithm (attention level) | GokEvolution effectively differentiated children with ADHD from TD children based on attentional scores. | 23 ADHD, 52 TD | 7-12 |
| Teo et al [82] | 2021 | Singapore | Clinic or lab | Experimental study (randomized controlled waitlist trial / pilot feasibility) | To examine the feasibility of a BCI program for children with ASD and co-occurring ADHD. | Frequency analysis | The BCI-based program showed greater improvement in the intervention group than in the waitlist control group on ADHD symptoms measured by the ADHD Rating Scale. | 20 ASD with ADHD (10 experimental, 10 control) | 8-12 |
| Bach-Morrow et al [49] | 2022 | USA | Clinic or lab | Experimental study (randomized controlled trial) | To evaluate whether EEG-based neurofeedback, FSCT, improves attention and executive functions. | Frequency analysis (Theta, alpha and Low beta, beta, TBR) | The FSCT group showed increased beta activity in the frontal cortex and improved attention and memory. | 46 ADHD (23 FSCT, 23 placebo) | 6-18 |
| Chang et al [50] | 2022 | Taiwan | Clinic or lab | Observational study (methodological study) | To develop EEG-based LSTM networks that use DL to learn cognitive state transitions and discriminate between ADHD and TD children. | Frequency analysis (PSD) | The ADHD group showed lower beta power at occipital sites, especially during cognitive tasks; the LSTM model achieved 90.5% accuracy in ADHD classification. | 30 ADHD, 30 TD | ADHD: 6.69 (±1.82), TD: 6.42 (±1.86) |
| Chen et al [51] | 2022 | Taiwan | Clinic or lab | Observational study (cross-sectional case-control) | To evaluate the validity of single and combined measurements—EEG, K-CPT-2, and DBDRS—for diagnosing ADHD. | Frequency analysis (PSD) | During the slow-rate task, the ADHD group showed increased delta power and decreased alpha and beta power at Cz and Pz sites. Multi-method combinations demonstrated superior diagnostic accuracy compared to any single measure. | 38 ADHD, 32 TD | 5-7 |
| Chen et al [52] | 2022 | Taiwan | Clinic or lab | Observational study (cross-sectional case-control) | To examine task-rate-related EEG dynamics and to identify neural biomarkers that differentiate among groups. | Frequency analysis (PSD) | The ADHD-H group showed increased central delta power and decreased beta power during slow-rate tasks, whereas the ADHD-L group showed elevated parietal delta and theta power and reduced alpha/beta power during fast-rate tasks. | 24 ADHD-H, 18 ADHD-L, 31 TD | 5-7 |
| Chen et al [84] | 2022 | Taiwan | Clinic or lab | Observational study (methodological study) | To investigate the FC profiles during rest and tasks of children with ADHD. | Connectivity analysis | PLVs may be the best FC indicators for classifying children with ADHD versus TD children. | 43 ADHD, 35 TD | 5-7 |
| Giannadou et al [53] | 2022 | United Kingdom | Home and clinic (or lab) | Observational study (methodological study) | To assess the feasibility and signal quality of home-based EEG and to explore children’s views on the experimental process. | Time analysis (ERP) | High-quality EEG signals were recorded at home using a gel-based system, with >97% showing reliable P1 and N1 ERPs comparable to lab settings. User feedback supported feasibility and guided ASC-specific protocol improvements. | 69 ASD | 6–15 |
| Jadavji et al [54] | 2022 | Canada | Home and clinic (or lab) | Observational study (program evaluation) | To assess the feasibility of a clinical, patient-centered BCI program specifically developed for children with severe motor impairments. | Frequency analysis, Time analysis (ERP) | Most children successfully used EEG-based BCI programs for communication and play, with strong family engagement. | 10 CP/Stroke with severe motor disabilities | 6-16 |
| Liao et al [55] | 2022 | China | Clinic or lab | Observational study (methodological study) | To develop a hybrid ML framework combining EEG, eye fixation, and facial expression data to improve early detection of ASD. | Frequency analysis (PSD) | The hybrid fusion ML model was able to distinguish ASD from TD with an accuracy of 87.5%, based on a weighted naive Bayes algorithm. | 40 ASD, 40 TD | 3-6 |
| Ramirez-Melendez et al [56] | 2022 | Spain | Clinic or lab | Experimental study (pilot randomized controlled trial) | To explore the usefulness of music as a tool for improving high-functioning ASD children's emotion recognition in facial expressions. | Frequency analysis | A multisensory emotional intervention combining facial expressions with congruent music enhanced emotion recognition and modulated arousal and valence responses. | 25 ASD (14 experimental, 11 control) | 6–11 |
| Westwood et al [57] | 2022 | United Kingdom | Clinic or lab | Experimental study (randomized controlled trial) | To examine the neuromodulatory effects of multi-session anodal tDCS combined with cognitive training over the right inferior frontal cortex. | Frequency analysis (PSD) | In 23 children and adolescents with ADHD, no significant clinical or cognitive effects were found on resting and Go/No-Go task-based QEEG measures. | 23 ADHD (13 tDCS, 10 control) | 10-18 |
| Dakwar-Kawar et al [58] | 2023 | Israel | Clinic or lab | Experimental study (randomized controlled trial) | To examine the effects of tRNS combined with cognitive training on ADHD symptoms and EEG markers. | Frequency analysis (FFT-based absolute spectral power in delta, theta, alpha, and beta) | tRNS combined with cognitive training improved ADHD symptoms and altered beta activity and E/I balance. | 23 ADHD (11 tRNS, 12 control) | 6–12 |
| Fan et al [59] | 2023 | Taiwan | Clinic or lab | Observational study (methodological study) | To improve ADHD diagnosis by combining EEG coherence and PSD during auditory (CATA) and visual tasks (CPT3). | Frequency analysis (PSD), connectivity analysis | The SVM model using CATA-based coherence and PSD features achieved an accuracy of 97.7% in classifying ADHD. | 30 ADHD, 14 TD | ADHD: 9.5 (±2.3), TD: 9.7 (±2.3) |
| Kawai et al [62] | 2023 | USA | Home | Observational study (cross-sectional case-control) | To compare sleep architecture (SWS and REM) between groups, and to assess whether sleep parameters relate to core ASD symptoms using a PSG device. | Frequency analysis (delta relative spectral power) | The ASD group showed increased SWS and a decreased REM ratio compared with the TD group. Higher SWS was associated with more severe restricted and repetitive behaviors in ASD. | 53 ASD, 66 TD | 4-18 |
| Lim et al [60] | 2023 | Singapore | Home and clinic (or lab) | Experimental study (randomized controlled trial) | To evaluate the feasibility and safety of a tablet-based neurofeedback game (Cogoland) at the child’s home. | Frequency analysis | Both home- and clinic-based groups showed comparable improvement in inattentive symptoms; EEG-driven attention feedback was effective and feasible without therapist support. | 20 ADHD (10 home-based, 10 clinic-based) | 6-12 |
| Luo et al [85] | 2023 | China | Home and clinic (or lab) | Experimental study (randomized controlled trial) | To compare the training outcomes of remotely delivered NFT, CCT, and combined NFT/CCT training in children with ADHD, and to evaluate the feasibility of home-based digital interventions. | Proprietary algorithm (attention level) | Remotely delivered NFT, CCT, and combined training all significantly improved ADHD symptoms, executive functions, and resting-state alpha power, with no significant differences in efficacy observed among the three intervention groups. | 80 ADHD | 7.1-12.3 |
| Lyu et al [61] | 2023 | China | Educational institution | Experimental study (case study) | To develop and assess a mobile AR neurofeedback game (Eggly) using EEG, aimed at enhancing social and communication skills. | Frequency analysis (PSD) | Eggly effectively improved social attention in children with ASD through real-time neurofeedback. A three-week study showed increasing attention index and engagement. | 5 ASD | 3-6 |
| McCabe et al [63] | 2023 | China | Educational institution | Observational study (cross-sectional case-control) | To examine frontal EEG power and activation differences among groups and investigate moderating effects of EDS. | Frequency analysis (PSD) | The ADHD-C group showed increased frontal delta/theta power compared with the ADHD-I and TD groups. EDS moderated alpha and beta power, particularly in the ADHD-C group. | 34 ADHD-C, 34 ADHD-I, 34 TD | 7-12 |
| Shehan et al [64] | 2023 | Sri Lanka | Not specified | Observational study (methodological study) | To detect ADHD levels in children using EEG and facial expression data via an ANN-CNN integrated system. | Time analysis | The ANN-CNN model using EEG and facial data achieved 94% accuracy in diagnosing ADHD. | Not reported (Children with and without ADHD) | 9-11 |
| Chen et al [65] | 2024 | Taiwan | Clinic or lab | Observational study (cross-sectional case-control) | To investigate the FC and complexity of brain activity among children with ADHD-F or ADHD-S cognitive processing speed, and TD children, during rest and task conditions. | Connectivity analysis | The ADHD-F group showed reduced long-range intra-hemispheric FC at rest. The ADHD-S group showed lower frontal inter-hemispheric FC and reduced frontal entropy during the task, indicating lower neural complexity. | 26 ADHD-F, 17 ADHD-S, 35 TD | 5-7 |
| Chen et al [96] | 2024 | Taiwan | Clinic or lab | Observational study (methodological study) | To develop a reliable multimodal AI system using EEG, K-CPT-2, DBDRS to assist ADHD diagnosis. | Proprietary algorithm (attention level) | The ensemble model combining EEG, CPT, and symptom scale achieved 97.4% accuracy, while the EEG-only model reached 95%. | 43 ADHD, 35 TD | 5-7 |
| Handayani et al [66] | 2024 | Indonesia | Not specified | Observational study (cross-sectional case-control) | To analyze differences in brain signal complexity between children with ASD and TD using MSE and explore associations with spectral power. | Complexity analysis (Multiscale entropy), Frequency analysis (PSD) | The ASD group showed lower MSE values than the TD group, indicating reduced brain complexity; spectral power was dominant in the low-frequency bands. | 7 ASD, 5 TD | 10-12 |
| Huang et al [67] | 2024 | Taiwan | Clinic or lab | Observational study (methodological study) | To facilitate early ADHD diagnosis in young children by analyzing EEG complexity and PSD features during rest and task. | Frequency analysis (PSD), Complexity analysis (entropy) | Combining beta power (O2) and fuzzy entropy (Pz) achieved 90% accuracy in classifying ADHD vs. TD using kNN. | 30 ADHD, 30 TD | 4–7 |
| Kaiser et al [86] | 2024 | Multi-countries(France, Spain, Switzrerland, Germany, and Belgium) | Home | Experimental study (randomized controlled trial) | To evaluate the utility of neurocognitive and executive functioning indices as treatment markers and predictive markers for both pharmacological and NF interventions in children and adolescents with ADHD. | Frequency analysis (TBR) | While neurocognitive indices are valuable treatment markers for monitoring methylphenidate effects, they offer limited prognostic value as predictive markers for ADHD treatment response compared to baseline symptom severity. | 146 ADHD (3:2 ratio for neurofeedback group:methylphenidate group) | 7–13 |
| Kwon et al [87] | 2024 | Korea | Home | Experimental study (randomized controlled trial) | To examine the effect of MNF training on the clinical symptoms, attention abilities, and executive functions of children with ADHD. | Frequency analysis (TBR) | MNF training can be used as an auxiliary treatment method for children with ADHD who do not show sufficient improvements with medication treatment. | 74 ADHD (35 experimental, 39 control) | 8–15 |
| Lin et al [68] | 2024 | Taiwan | Clinic or lab | Observational study (cross-sectional case-control) | To investigate FC differences between groups during visual (CPT) and auditory (CATA) attention tasks. | Connectivity analysis | The ADHD group showed reduced alpha-band FC during the CATA task, especially in the temporal lobe. Overall, CATA elicited more distinct FC differences than CPT across all frequency bands. | 53 ADHD, 19 TD | 8-16 |
| Rebollo et al [89] | 2024 | Spain | Clinic or lab | Experimental study (randomized controlled trial) | To compare paper-based versus gamified mobile learning in ADHD by measuring differences in cognitive/emotional engagement and EEG-derived brain activity—and to test whether EEG indicators align with children’s self-reported learning experiences. | Proprietary algorithm (level of attention, relaxation, appreciation, mental effort and familiarity) | Gamified learning improved attention and emotional engagement, and EEG showed greater mental effort and appreciation during gameplay. | 7 ADHD | 6-9 |
| Tseng et al [69] | 2024 | Taiwan | Not specified | Observational study (methodological study) | To develop an assessment method for identifying ASD using a game-based social cognitive paradigm that captures both local brain activation and FC. | Time analysis (ERP), time–frequency analysis (ERSP), connectivity analysis (PLI) | Reduced theta synchronization in the ASD group and lower LPP amplitudes and larger P200 amplitudes in autistic preadolescents and teenagers suggested that social interaction difficulties in autism were linked to specific brain activation patterns. | 12 ASD, 12 TD | ASD: 14.71 (± 2.92), TD: 11.83 (± 1.27) |
| Tseng et al [70] | 2024 | Taiwan | Clinic or lab | Observational study (cross-sectional case-control) | To evaluate gaze-following and associated brain activity, aiming to identify cognitive biomarkers for early intervention using game-based EEG. | Connectivity analysis (PLI) | Autistic children showed decreased theta synchronization and FC and increased alpha and beta desynchronization. | 12 ASD, 18 TD | ASD: 4.75 (±1.76), TD: 4.89 (±1.24) |
| Wang et al [71] | 2024 | China | Clinic or lab | Experimental study (randomized controlled trial) | To evaluate the effect of wearable mu rhythm EEG neurofeedback on social communication. | Frequency analysis (Mu-rhythm) | The neurofeedback group with active mu rhythm NFT showed significantly greater improvements in expressive language and cognitive awareness. | 41 ASD (17 NFT, 24 placebo) | 3-6 |
| Cai et al [72] | 2025 | China | Clinic or lab | Observational study (methodological study) | To investigate FC differences under emotional stimuli and evaluate EEG-based classification performance for ASD. | Connectivity analysis (coherence, PLV, PLI, WPLI) | FC under positive and negative emotional clips achieved a classification accuracy exceeding 85%. | 32 ASD, 32 TD | 2-7 |
| Huang et al [88] | 2025 | Taiwan | Clinic or lab | Observational study (methodological study) | To combine EEG (resting and attention-testing state) and a language model to assess ADHD. | Frequency analysis (PSD) | Slow-rate EEG features combined with XGBoost achieved superior ADHD classification performance, and integrating RAG provided clinically interpretable explanations beyond conventional EEG-based classifiers. | 17 ADHD, 11 TD | 8-17 |
| Kim et al [95] | 2025 | South Korea | Home | Experimental study (randomized controlled trial) | To investigate the therapeutic effects of theta/beta MNF on clinical outcomes and TGC in children with ADHD. | Frequency analysis (TBR) | MNF significantly increased TGC in children with ADHD, which correlated with a reduction in ADHD symptoms (ARS scores). | 78 ADHD, 61 TD | 8-15 |
| Mandapati and Ranjan [73] | 2025 | India | Educational institution | Experimental study (non-randomized controlled trial) | To examine the effects of VR-based AVE on learning in ADHD children. | Frequency analysis (PSD) | 72% of participants showed improved attention and cognitive ability after AVE sessions. | 11 ADHD | 6-12 |
| Mercado-Aguirre et al [91] | 2025 | Columbia | Not specified | Observational study (methodological study) | To evaluate ERP (odd-ball task) signals as classifiers of ADHD. | Time analysis (ERP), Frequency analysis (spectral power), Complexity analysis | SVM/Decision Trees classified ADHD vs controls from auditory ERP EEG with 86.36% accuracy and 95.45% sensitivity, supporting portable EEG as an objective tool for early diagnosis. | 22 ADHD, 25 TD | 7-17 |
| Pang et al [90] | 2025 | China | Clinic or lab | Observational study (pilot validation / case-control) | To develop a portable multi-modal data acquisition platform for ASD assessment and conduct a pilot validation of its effectiveness in early screening. | Frequency analysis (PSD), Complexity analysis (entropy) | The platform identified significant EEG differences in children with ASD, including a 226% increase in delta-band power (p = 0.0015) and a 38.5% reduction in approximate entropy (p < 0.0001), compared with TD controls. | 3 ASD, 4 TD | 6-9 |
| Penmetsa et al [92] | 2025 | USA | Clinic or lab | Observational study (methodological study) | To characterize task-related EEG-based cognitive/attentional dynamics in ASD and to build classifiers that distinguish ASD from TD. | Frequency analysis (PSD), Time–frequency analysis (Morlet wavelet, dynamic decibel-normalized power) | Children with ASD showed distinct EEG band-power and time–frequency patterns during STEM-related cognitive tasks, and ML models were able to classify ASD versus TD children with high accuracy using these EEG features. | 3 ASD, 7 TD | 4-13 |
| Wang et al [93] | 2025 | China | Clinic or lab | Observational study (methodological study) | To develop and validate a portable EEG-based stimulation paradigm that objectively detects tactile processing abnormalities and enables classification of ASD versus TD. | Time analysis (ERP) | ASD children exhibited prolonged but less efficient tactile discrimination and compensatory prefrontal activation, achieving 85.2% classification accuracy. | 36 ASD, 36 TD | 1-5 |
| Dakwar-Kawar et al [74] | 2026 | Israel | Clinic or lab | Experimental study (randomized controlled trial) | To examine behavioral and neural differences between children with and without ADHD and to evaluate the immediate and long-term effects of a combined tRNS and cognitive training intervention on modulating neural activity and improving symptoms in children with ADHD. | Time analysis (ERP), Time–frequency analysis (wavelet-based ERSP), Source-space analysis (RSBL-based cortical/network-level analysis) | Children with ADHD exhibited elevated aperiodic exponents, which were significantly reduced through combined tRNS and cognitive training, and these neural changes were associated with improvements in clinical symptoms, suggesting a partial mediation effect. | 23 ADHD (11 tRNS+CT vs 12 Sham+CT), 33 TD | 6-12 |
| Ezedinma et al [94] | 2026 | Australia | Home | Observational study | To evaluate the feasibility, adequacy, and cost-effectiveness of home-based Level 2 PSG in children with ASD. | Time analysis (sleep staging, Latency) | Home-based Level 2 PSG is feasible, technically adequate, and cost-effective for repeated sleep assessment in children with ASD during an interventional trial. | 20 ASD | 6-12 |

*ADHD, attention-deficit/hyperactivity disorder; ADHD-C, ADHD combined presentation; ADHD-F, ADHD exhibiting fast; ADHD-H, ADHD hyperactive-impulsive presentation / ADHD exhibiting high cognitive proficiency; ADHD-I, ADHD inattentive presentation; ADHD-L, ADHD exhibiting low cognitive proficiency; ADHD-S, ADHD exhibiting slow; AI, artificial intelligence; ANNs, artificial neural networks; AR, augmented reality; ARS, anticholinergic risk scale; ASC, autism spectrum conditions; ASD, autism spectrum disorders; AVE, audiovisual brainwave entrainment; BCI, brain–computer interface; CATA, Continuous Auditory Test of Attention; CCT, computerized cognitive training; CNN, cellular neural network; CNNs, convolutional neural networks; CP, cerebral palsy; CPT, Continuous Performance Test; CT, computed tomography; DAT, Dolphin-Assisted Therapy; DBDRS, Disruptive Behavior Disorder Rating Scale; DL, deep learning; ECG, electrocardiogram; EDS, excessive daytime sleepiness; EEG, electroencephalography / electroencephalogram; E/I, excitation/inhibition; EO, eyes-open; ERP, event-related potential; ERSP, event-related spectral perturbation; FC, functional connectivity; FFT-based PSD, fast fourier transformer–based power spectral density; FO, focus; FSCT, Frequency-Specific Cognitive Training; ID, intellectual disability; ISCP, infantile spastic cerebral palsy; kNN, k-Nearest Neighbors; K-CPT, Conners' Kiddie Continuous Performance Test; K-CPT-2, Conners’ Kiddie Continuous Performance Test Second Edition; LD, learning disorder; LSTM, long short-term memory; ML, machine learning; MNF, mobile neurofeedback; MSE, multiscale entropy; N/S, Not specified; NCAT, neurocognitive assessment tool; NF, non-pharmacological; NFT, neurofeedback training; OCD, obsessive compulsive disorder; PLI, phase lag index; PLV, phase-locking value; PSD, power spectral density; PSG, polysomnography; RAG, retrieval-augmented generation; REM, rapid eye movement; RSBL, random subspace binary logic; SLD, specific learning disorder; SNAP-IV, the Swanson, Nolan, and Pelham rating scale version IV; STFT, short-time fourier transform; SVM, support vector machine; SWS, slow-wave sleep; tDCS, transcranial direct current stimulation; tRNS, transcranial random noise stimulation; TBR, Theta/Beta ratio; TD, typical development; TGC, theta-gamma coupling; VR, virtual reality; WPLI, weighted phase lag index.

**References**

33. Modarres-Zadeh M, Xu X, Jaromczyk A, Senders S. A neuro-behavioral test and algorithms for screening and evaluating therapy in ADHD. Conf Proc IEEE Eng Med Biol Soc. 2005;2005:2486-2489. [doi: 10.1109/IEMBS.2005.1616973]

34. Iramina K, Matsuda K, Ide J, Noguchi Y. Monitoring system of neuronal activity and moving activity without restraint using wireless EEG, NIRS and accelerometer. Presented at: 2010 IEEE EMBS Conference on Biomedical Engineering and Sciences (IECBES); Nov 30 to Dec 2, 2010; Kuala Lumpur, Malaysia. 2010. [doi: 10.1109/IECBES.2010.5742285]

35. Israsena P, Hemrungrojn S, Sukwattanasinit N, Maes M. Development and evaluation of an interactive electro-encephalogram-based neurofeedback system for training attention and attention defects in children. J Med Imaging Hlth Inform. Sep 1, 2015;5(5):1045-1052. [doi: 10.1166/jmihi.2015.1492]

36. Zennifa F, Ide J, Noguchi Y, Iramina K. Monitoring of cognitive state on mental retardation child using EEG, ECG and NIRS in four years study. Presented at: 2015 37th Annual International Conference of the IEEE Engineering in Medicine and Biology Society (EMBC); Nov 5, 2015; Milan, Italy. 2015. [doi: 10.1109/EMBC.2015.7319908]

37. Billeci L, Tonacci A, Tartarisco G, et al. An integrated approach for the monitoring of brain and autonomic response of children with autism spectrum disorders during treatment by wearable technologies. Front Neurosci. 2016;10(276):276. [doi: 10.3389/fnins.2016.00276] [Medline: 27445652]

38. Martínez F, Barraza C, González N, González J. KAPEAN: understanding affective states of children with ADHD. J Educ Technol Soc. 2016;19(2):18-28. URL: https://www.jstor.org/stable/jeductechsoci.19.2.18

39. Perales F, Amengual E. Combining EEG and serious games for attention assessment of children with cerebral palsy. Converging Clinical and Engineering Research on Neurorehabilitation II. Biosystems & Biorobotics, vol 15. Cham: Springer; 2017. [doi: 10.1007/978-3-319-46669-9_66]

40. Askari E, Setarehdan SK, Sheikhani A, Mohammadi MR, Teshnehlab M. Modeling the connections of brain regions in children with autism using cellular neural networks and electroencephalography analysis. Artif Intell Med. Jul 2018;89:40-50. [doi: 10.1016/j.artmed.2018.05.003] [Medline: 30007788]

41. Eroglu G, Aydin S, Cetin M, Balcisoy S. Improving cognitive functions of dyslexies using multi-sensory learning and EEG neurofeedback. Presented at: 2018 26th Signal Processing and Communications Applications Conference (SIU); May 2-5, 2018; Izmir. 2018. [doi: 10.1109/SIU.2018.8404711]

42. Mercado-Aguirre IM, Gutiérrez-Ruiz K, Contreras-ortiz SH. Acquisition and analysis of cognitive evoked potentials using an emotiv headset for ADHD evaluation in children. Presented at: 2019 XXII Symposium on Image, Signal Processing and Artificial Vision (STSIVA); Apr 24-26, 2019; Bucaramanga, Colombia. 2019. [doi: 10.1109/STSIVA.2019.8730225]

43. Chu KC, Lu HK, Huang MC, et al. Using mobile electroencephalography and actigraphy to diagnose attention-deficit/hyperactivity disorder: case-control comparison study. JMIR Ment Health. Jun 19, 2020;7(6):e12158. [doi: 10.2196/12158] [Medline: 32558658]

44. Liao CY, Chen RC, Tai SK. Children with attention deficit hyperactivity disorder and learning disability detection via brainwave measurements. ICIC Express Lett B: Appl. 2020;11:51-58. [doi: 10.24507/icicelb.11.01.5]

45. Matamoros OM, Escobar JJM, Tejeida Padilla R, Lina Reyes I. Neurodynamics of patients during a dolphin-assisted therapy by means of a fractal intraneural analysis. Brain Sci. Jun 25, 2020;10(6):403. [doi: 10.3390/brainsci10060403] [Medline: 32630512]

46. Chen IC, Chang CH, Chang Y, Lin DS, Lin CH, Ko LW. Neural dynamics for facilitating ADHD diagnosis in preschoolers: central and parietal delta synchronization in the Kiddie continuous performance test. IEEE Trans Neural Syst Rehabil Eng. 2021;29(1524–33):1524-1533. [doi: 10.1109/TNSRE.2021.3097551] [Medline: 34280103]

47. Peng S, Xu R, Yi X, Hu X, Liu L, Liu L. Early screening of children with autism spectrum disorder based on electroencephalogram signal feature selection with L1-Norm regularization. Front Hum Neurosci. 2021;15:656578. [doi: 10.3389/fnhum.2021.656578] [Medline: 34239427]

48. Serrano-Barroso A, Siugzdaite R, Guerrero-Cubero J, et al. Detecting attention levels in ADHD children with a video game and the measurement of brain activity with a single-channel BCI headset. Sensors (Basel). May 6, 2021;21(9):3221. [doi: 10.3390/s21093221] [Medline: 34066492]

49. Bach-Morrow L, Boccalatte F, DeRosa A, et al. Functional changes in prefrontal cortex following frequency-specific training. Sci Rep. Nov 24, 2022;12(1):20316. [doi: 10.1038/s41598-022-24088-7] [Medline: 36434008]

50. Chang Y, Stevenson C, Chen IC, Lin DS, Ko LW. Neurological state changes indicative of ADHD in children learned via EEG-based LSTM networks. J Neural Eng. Feb 10, 2022;19(1). [doi: 10.1088/1741-2552/ac4f07] [Medline: 35081524]

51. Chen IC, Lee PW, Wang LJ, Chang CH, Lin CH, Ko LW. Incremental validity of multi-method and multi-informant evaluations in the clinical diagnosis of preschool ADHD. J Atten Disord. Aug 2022;26(10):1293-1303. [doi: 10.1177/10870547211045739] [Medline: 34949123]

52. Chen IC, Chen CL, Chang CH, et al. Task-rate-related neural dynamics using wireless EEG to assist diagnosis and intervention planning for preschoolers with ADHD exhibiting heterogeneous cognitive proficiency. J Pers Med. Apr 30, 2022;12(5):731. [doi: 10.3390/jpm12050731] [Medline: 35629153]

53. Giannadou A, Jones M, Freeth M, Samson AC, Milne E. Investigating neural dynamics in autism spectrum conditions outside of the laboratory using mobile electroencephalography. Psychophysiology. Apr 2022;59(4):e13995. [doi: 10.1111/psyp.13995] [Medline: 34982474]

54. Jadavji Z, Zewdie E, Kelly D, Kinney-Lang E, Robu I, Kirton A. Establishing a clinical brain-computer interface program for children with severe neurological disabilities. Cureus. Jun 2022;14(6):e26215. [doi: 10.7759/cureus.26215] [Medline: 35891842]

55. Liao M, Duan H, Wang G. Application of machine learning techniques to detect the children with autism spectrum disorder. J Healthc Eng. 2022;2022(1):9340027. [doi: 10.1155/2022/9340027] [Medline: 35368925]

56. Ramirez-Melendez R, Matamoros E, Hernandez D, Mirabel J, Sanchez E, Escude N. Music-enhanced emotion identification of facial emotions in autistic spectrum disorder children: a pilot EEG study. Brain Sci. May 30, 2022;12(6):704. [doi: 10.3390/brainsci12060704] [Medline: 35741590]

57. Westwood SJ, Bozhilova N, Criaud M, et al. The effect of transcranial direct current stimulation (tDCS) combined with cognitive training on EEG spectral power in adolescent boys with ADHD: a double-blind, randomized, sham-controlled trial. IBRO Neurosci Rep. Jun 2022;12:55-64. [doi: 10.1016/j.ibneur.2021.12.005] [Medline: 35746969]

58. Dakwar-Kawar O, Mairon N, Hochman S, Berger I, Cohen Kadosh R, Nahum M. Transcranial random noise stimulation combined with cognitive training for treating ADHD: a randomized, sham-controlled clinical trial. Transl Psychiatry. Aug 2, 2023;13(1):271. [doi: 10.1038/s41398-023-02547-7] [Medline: 37528107]

59. Fan ZC, Lin RW, Tsai CS, et al. Unveiling multivariate EEG features: a novel approach to enhancing ADHD diagnosis through visual and auditory attention tests. Presented at: 2023 International Conference on Fuzzy Theory and Its Applications (iFUZZY); Oct 26-29, 2023; Penghu, Taiwan. 2023. [doi: 10.1109/iFUZZY60076.2023.10324282]

60. Lim CG, Soh CP, Lim SSY, Fung DSS, Guan C, Lee TS. Home-based brain-computer interface attention training program for attention deficit hyperactivity disorder: a feasibility trial. Child Adolesc Psychiatry Ment Health. Jan 25, 2023;17(1):15. [doi: 10.1186/s13034-022-00539-x] [Medline: 36698168]

61. Lyu Y, An P, Xiao Y, et al. Eggly: designing mobile augmented reality neurofeedback training games for children with autism spectrum disorder. Proc ACM Interact Mob Wearable Ubiquitous Technol. 2023;7:1-29. [doi: 10.1145/3596251]

62. Kawai M, Buck C, Chick CF, et al. Sleep architecture is associated with core symptom severity in autism spectrum disorder. Sleep. Mar 9, 2023;46(3):zsac273. [doi: 10.1093/sleep/zsac273] [Medline: 36385326]

63. McCabe LE, Johnstone SJ, Jiang H, Sun L, Zhang DW. Links between excessive daytime sleepiness and EEG power and activation in two subtypes of ADHD. Biol Psychol. Feb 2023;177:108504. [doi: 10.1016/j.biopsycho.2023.108504] [Medline: 36681294]

64. Shehan A, Anjali S, Sanjula L, Lavanya M, Thelijjagoda S, Krishara J. Neuroassist ADHD analyzer: a smart application for recognizing attention deficit hyperactivity disorder (ADHD) levels in children. Presented at: 2023 International Conference on Innovative Computing, Intelligent Communication and Smart Electrical Systems (ICSES); Dec 14-15, 2023; Chennai, India. 2023. [doi: 10.1109/ICSES60034.2023.10465399]

65. Chen IC, Chang CL, Huang IW, Chang MH, Ko LW. Electrophysiological functional connectivity and complexity reflecting cognitive processing speed heterogeneity in young children with ADHD. Psychiatry Res. Oct 2024;340:116100. [doi: 10.1016/j.psychres.2024.116100] [Medline: 39121760]

66. Handayani N, Asyrafi H, Khotimah SN. EEG complexity in children with autism spectrum disorders: a multiscale entropy analysis. AIP Conf Proc. Aug 28, 2024;3210(1). [doi: 10.1063/5.0228307]

67. Huang IW, Jheng YC, Chen IC, Ko LW. Complexity analysis based on parietal fuzzy entropy to facilitate ADHD diagnosis in young children. Presented at: Annu Int Conf IEEE Eng Med Biol Soc; Jul 15-19, 2024; Orlando, FL, USA. 2024. [doi: 10.1109/EMBC53108.2024.10781919]

68. Lin JW, Fan ZC, Tzou SC, Wang LJ, Ko LW. Temporal alpha dissimilarity of ADHD brain network in comparison with CPT and CATA. IEEE Trans Neural Syst Rehabil Eng. 2024;32:1333-1343. [doi: 10.1109/TNSRE.2024.3360137] [Medline: 38289841]

69. Tseng YL, Lee CH, Chiu YN, et al. Characterizing autism spectrum disorder through fusion of local cortical activation and global functional connectivity using game-based stimuli and a mobile EEG system. IEEE Trans Neural Syst Rehabil Eng. 2024;32:3026-3035. [doi: 10.1109/TNSRE.2024.3417210] [Medline: 39163173]

70. Tseng YL, Liu HH, Chiu YN, et al. Electroencephalography connectivity assesses cognitive disorders of autistic children during game-based social interaction. IEEE Trans Cogn Dev Syst. 2024;16(2):782-793. [doi: 10.1109/TCDS.2023.3297609]

71. Wang XN, Zhang T, Han BC, et al. Wearable EEG neurofeedback based-on machine learning algorithms for children with autism: a randomized, placebo-controlled study. Curr Med Sci. Dec 2024;44(6):1141-1147. [doi: 10.1007/s11596-024-2938-3]

72. Cai C, Wang J, Lin J, et al. Functional connectivity analysis of children with autism under emotional clips. IEEE Trans Affective Comput. 2025;16(3):1646-1659. [doi: 10.1109/TAFFC.2025.3528920]

73. Mandapati M, Ranjan P. Virtual reality based audio visual brainwave entrainment to improve learning in children with attention deficit hyperactive disorder. Appl Neuropsychol Child. Jan 23, 2025:1-15. [doi: 10.1080/21622965.2025.2455102] [Medline: 39847472]

74. Dakwar-Kawar O, Francis AJA, Arya R, et al. The effects of transcranial random noise stimulation on excitation/inhibition balance in ADHD. Neuroimage Clin. 2026;49:103923. [doi: 10.1016/j.nicl.2025.103923] [Medline: 41496381] [Epub 2025 Dec 5]

75. Lim CG, Lee TS, Guan C, et al. A brain-computer interface based attention training program for treating attention deficit hyperactivity disorder. PLoS One. 2012;7(10):e46692. [doi: 10.1371/journal.pone.0046692] [Medline: 23115630]

76. Jiang H, Johnstone SJ. A preliminary multiple case report of neurocognitive training for children with AD/HD in China. Sage Open. Apr 1, 2015;5(2):2158244015586811. [doi: 10.1177/2158244015586811]

77. Garzotto F, Gelsomini M, Pappalardo A, Sanna C, Stella E, Zanella M. Using brain signals in adaptive smart spaces for disabled children. Presented at: Proceedings of the 2016 CHI Conference Extended Abstracts on Human Factors in Computing Systems; May 7-12, 2016; San Jose, California, USA. 2016. [doi: 10.1145/2851581.2892533]

78. Qian X, Loo BRY, Castellanos FX, et al. Brain-computer-interface-based intervention re-normalizes brain functional network topology in children with attention deficit/hyperactivity disorder. Transl Psychiatry. Aug 10, 2018;8(1):149. [doi: 10.1038/s41398-018-0213-8] [Medline: 30097579]

79. Fan J, Wade JW, Key AP, Warren ZE, Sarkar N. EEG-based affect and workload recognition in a virtual driving environment for ASD intervention. IEEE Trans Biomed Eng. Jan 2018;65(1):43-51. [doi: 10.1109/TBME.2017.2693157] [Medline: 28422647]

80. Mercado J, Espinosa-Curiel I, Escobedo L, Tentori M. Developing and evaluating a BCI video game for neurofeedback training: the case of autism. Multimed Tools Appl. May 2019;78(10):13675-13712. [doi: 10.1007/s11042-018-6916-2]

81. Lim CG, Poh XWW, Fung SSD, et al. A randomized controlled trial of a brain-computer interface based attention training program for ADHD. PLoS One. 2019;14(5):e0216225. [doi: 10.1371/journal.pone.0216225] [Medline: 31112554]

82. Teo SHJ, Poh XWW, Lee TS, et al. Brain-computer interface based attention and social cognition training programme for children with ASD and co-occurring ADHD: a feasibility trial. Res Autism Spectr Disord. Nov 2021;89:101882. [doi: 10.1016/j.rasd.2021.101882]

83. Johnstone SJ, Parrish L, Jiang H, Zhang DW, Williams V, Li S. Aiding diagnosis of childhood attention-deficit/hyperactivity disorder of the inattentive presentation: discriminant function analysis of multi-domain measures including EEG. Biol Psychol. Apr 2021;161:108080. [doi: 10.1016/j.biopsycho.2021.108080] [Medline: 33744372]

84. Chen IC, Chang CL, Chang MH, Ko LW. Atypical functional connectivity during rest and task-related dynamic alteration in young children with attention deficit hyperactivity disorder: an analysis using the phase-locking value. Psychiatry Clin Neurosci. Jun 2022;76(6):235-245. [doi: 10.1111/pcn.13344] [Medline: 35235255]

85. Luo X, Guo X, Zhao Q, et al. A randomized controlled study of remote computerized cognitive, neurofeedback, and combined training in the treatment of children with attention-deficit/hyperactivity disorder. Eur Child Adolesc Psychiatry. Aug 2023;32(8):1475-1486. [doi: 10.1007/s00787-022-01956-1]

86. Kaiser A, Aggensteiner PM, Blasco Fontecilla H, et al. Limited usefulness of neurocognitive functioning indices as predictive markers for treatment response to methylphenidate or neurofeedback@home in children and adolescents with ADHD. Front Psychiatry. 2024;14:1331004. [doi: 10.3389/fpsyt.2023.1331004]

87. Kwon SY, Seo G, Jang M, et al. The effect of mobile neurofeedback training in children with attention deficit hyperactivity disorder: a randomized controlled trial. Clin Psychopharmacol Neurosci. Feb 29, 2024;22(1):67-78. [doi: 10.9758/cpn.23.1054] [Medline: 38247413]

88. Huang TC, Yang SR, Chiang SE, He C, Lin DS, Ko LW. EEG slow-rate task features with retrieval-augmented generation for ADHD classification in school-age children. Presented at: 2025 International Automatic Control Conference (CACS); Nov 5-8, 2025; Hsinchu, Taiwan. [doi: 10.1109/CACS67552.2025.11288161]

89. Rebollo C, Tonda A, Sorlí C, Remolar I. Brain waves and learning in children with ADHD: impact of educational video games vs traditional methods. Presented at: 21st International Conference on Cognition and Exploratory Learning in Digital Age (CELDA 2024); Oct 26-28, 2024; Zagreb, Croatia. URL: https://eric.ed.gov/?id=ED665389

90. Pang L, Zhao M, Ma C, et al. A multimodal data-driven assessment system for autism spectrum disorder in children: development and pilot validation of a multimodal acquisition platform. Presented at: 2025 47th Annual International Conference of the IEEE Engineering in Medicine and Biology Society (EMBC); Jul 14-18, 2025; Copenhagen, Denmark. [doi: 10.1109/EMBC58623.2025.11254484]

91. Mercado-Aguirre I, Gutiérrez-Ruiz K, Contreras-Ortiz SH. Classification of auditory ERPs for ADHD detection in children. J Med Eng Technol. Apr 2025;49(3):69-78. [doi: 10.1080/03091902.2025.2477506] [Medline: 40117461]

92. Penmetsa H, Abbasi R, Yellamilli N, et al. Investigating brain activity of children with autism spectrum disorder during STEM-related cognitive tasks. Information. 2025;16(10):880. [doi: 10.3390/info16100880]

93. Wang W, Liu Y, Shi P, et al. Altered tactile abnormalities in children with ASD during tactile processing and recognition revealed by dynamic EEG features. Front Psychiatry. 2025;16:1611438. [doi: 10.3389/fpsyt.2025.1611438] [Medline: 41035957]

94. Ezedinma U, Burgess S, Greenhill J, et al. Home polysomnography in children with autism spectrum disorder: a prospective observational study. J Sleep Res. Aug 2026;35(4):e70265. [doi: 10.1111/jsr.70265] [Medline: 41392642] [Epub 2025 Dec 15]

95. Kim JW, Kim BN, Kim JI, Yang CM, Kwon J. Effects of mobile neurofeedback on theta–gamma coupling and clinical outcomes in children with ADHD: a double-blind, sham-controlled randomized clinical trial. Neuropsychiatr Dis Treat. 2025;21:2733-2746. [doi: 10.2147/NDT.S554756] [Medline: 41368620]

96. Chen IC, Chang CL, Chang MH, Ko LW. The utility of wearable electroencephalography combined with behavioral measures to establish a practical multi-domain model for facilitating the diagnosis of young children with attention-deficit/hyperactivity disorder. J Neurodev Disord. Nov 11, 2024;16(1):62. [doi: 10.1186/s11689-024-09578-1] [Medline: 39528958]
