# Supplementary material for: Wireless Electroencephalography in Research on Children With Developmental Disabilities: Scoping Review
Source: J Med Internet Res. 2026 Aug 25;28:e84707. doi: 10.2196/84707 (PMC13505603; doi:10.2196/84707)
Supplement: Supplementary file 3 [file jmir-v28-e84707-s003.docx]

**Technical specifications of all wireless electroencephalography devices identified across 65 study–device pairs in the reviewed studies.**

| Study [Reference] | Year | Model, Manufacturer | Wireless Transmission | Channels | Electrode Type | Device Design | Sampling Rate (Hz) | CoME-D^a^ | | Regulatory Status^b^ | EEG Application Purpose^c^ |
| --- | --- | --- | --- | --- | --- | --- | --- | --- | --- | --- | --- |
| Modarres-Zadeh et al [33] | 2005 | Crystal 16, Cleveland Medical Devices | 902-928 MHz telemetry | 8 | Gel | NR | 200 | NR | Medical device (FDA-cleared) | | Biomarker-driven clinical assessment and diagnosis |
| Iramina et al [34] | 2010 | Custom Wireless EEG | 315-322 MHz telemetry | 2 | NR | NR | 400 | 2 | Research use only | | Task/state monitoring |
| Lim et al [75] | 2012 | Wireless EEG developed by Neurosky | 0 | 2 | NR | NR | NR | NR | Consumer-grade | | BCI technology |
| Israsena et al [35] | 2015 | Mindwave, Neurosky | Bluetooth, BLE | 1 | Dry | Headset | 512 | 4 | Consumer-grade | | BCI technology |
| Jiang and Johnstone [76] | 2015 | Mindwave, Neurosky | Bluetooth, BLE | 1 | Dry | Headset | 512 | 4 | Consumer-grade | | BCI technology |
| Zennifa et al [36] | 2015 | Custom Wireless EEG | 315-322 MHz telemetry | 2 | NR | NR | 400 | 2 | Research use only | | Intervention effect evaluation |
| Billeci et al [37] | 2016 | Enobio, Neuroelectrics | Wi-Fi | 19 | Dry | Cap | 500 | 3 | Medical device (FDA-cleared) | | Task/state monitoring |
| Garzotto et al [77] | 2016 | Mindwave, Neurosky | Bluetooth, BLE | 1 | Dry | Headset | 512 | 4 | Consumer-grade | | Task/state monitoring |
| Martínez et al [38] | 2016 | Epoc, Emotiv | Bluetooth, BLE, proprietary | 14 | Semi-dry (saline) | Headset | NR | 4 | Consumer-grade | | Task/state monitoring |
| Perales et al [39] | 2017 | Enobio, Neuroelectrics | Wi-Fi | 8 | NR | Cap | 500 | 3 | Medical device (FDA-cleared) | | Biomarker-driven clinical assessment and diagnosis |
| Askari et al [40] | 2018 | Epoc, Emotiv | Bluetooth, BLE, proprietary | 14 | Semi-dry (saline) | Headset | 128 | 4 | Consumer-grade | | Biomarker-driven clinical assessment and diagnosis |
| Eroğlu et al [41] | 2018 | Epoc, Emotiv | Bluetooth, BLE, proprietary | 14 | Semi-dry (saline) | Headset | 128 | 4 | Consumer-grade | | BCI technology |
| Fan et al [79] | 2018 | Epoc, Emotiv | Bluetooth, BLE, proprietary | 14 | Semi-dry (saline) | Headset | 128 | 4 | Consumer-grade | | Task/state monitoring |
| Qian et al [78] | 2018 | Wireless EEG developed by Zeo | Bluetooth | 2 | Dry | Headband | 128 | 3 | Consumer-grade | | BCI technology |
| Lim et al [81] | 2019 | Not specified | Bluetooth | 2 | Dry | Headband | NR | 4 | Research use only | | BCI technology |
| Mercado et al [80] | 2019 | BrainLink, Macrotellect | Bluetooth, BLE | 2 | Dry | Headset | 512 | 4 | Consumer-grade | | BCI technology |
| Mercado-Aguirre et al [42] | 2019 | Epoc, Emotiv | Bluetooth, BLE, proprietary | 14 | Semi-dry (saline) | Headset | NR | 4 | Consumer-grade | | Biomarker-driven clinical assessment and diagnosis |
| Chu et al [43] | 2020 | MindSet, NeuroSky | Bluetooth, BLE | 1 | Dry | Headset | 512 | 4 | Consumer-grade | | Biomarker-driven clinical assessment and diagnosis |
| Liao et al [44] | 2020 | Mindwave, Neurosky | Bluetooth, BLE | 1 | Dry | Headset | 512 | 4 | Consumer-grade | | Biomarker-driven clinical assessment and diagnosis |
| Matamoros et al [45] | 2020 | Mindwave, Neurosky | Bluetooth, BLE | 1 | Dry | Headset | 512 | 4 | Consumer-grade | | Intervention effect evaluation |
| Chen et al [46] | 2021 | BR 8, The Brain Research Center of National Chiao Tung University | Bluetooth | 8 | Semi-dry (saline) | Headset | 1000 | 3 | Research use only | | Biomarker-driven clinical assessment and diagnosis |
| Johnstone et al [83] | 2021 | Mindwave, Neurosky | Bluetooth, BLE | 1 | Dry | Headset | 512 | 4 | Consumer-grade | | Biomarker-driven clinical assessment and diagnosis |
| Peng et al [47] | 2021 | Epoc, Emotiv | Bluetooth, BLE, proprietary | 14 | Semi-dry (saline) | Headset | NR | 4 | Consumer-grade | | Biomarker-driven clinical assessment and diagnosis |
| Serrano-Barroso et al [48] | 2021 | Mindwave, Neurosky | Bluetooth, BLE | 1 | Dry | Headset | 512 | 4 | Consumer-grade | | BCI technology |
| Teo et al [82] | 2021 | Not specified | Bluetooth | 2 | Dry | Headband | NR | 4 | Research use only | | BCI technology |
| Bach-Morrow et al [49] | 2022 | THINK headset, Think Interfaces | Bluetooth | 2 | Dry | Headset | NR | 3 | Consumer-grade | | BCI technology |
| Chang et al [50] | 2022 | BR 8, The Brain Research Center of National Chiao Tung University | Bluetooth | 8 | Semi-dry (saline) | Headset | 512 | 3 | Research use only | | Biomarker-driven clinical assessment and diagnosis |
| Chen et al [51] | 2022 | BR 8, The Brain Research Center of National Chiao Tung UniversityNational Chiao Tung University | Bluetooth | 8 | Semi-dry (saline) | Headset | 1000 | 3 | Research use only | | Biomarker-driven clinical assessment and diagnosis |
| Chen et al [52] | 2022 | BR 8, The Brain Research Center of National Chiao Tung University | Bluetooth | 8 | Semi-dry (saline) | Headset | 1000 | 3 | Research use only | | Biomarker-driven clinical assessment and diagnosis |
| Chen et al [84] | 2022 | BR 8, The Brain Research Center of National Chiao Tung University | Bluetooth | 8 | Semi-dry (saline) | Headset | 1000 | 3 | Research use only | | Biomarker-driven clinical assessment and diagnosis |
| Giannadou et al [53] | 2022 | Eego™ sports, ANTneuro | Wi-Fi, Bluetooth | 32 | Gel | Cap | 2000 | 3 | Medical device (FDA-cleared) | | Biomarker-driven clinical assessment and diagnosis |
| Jadavji et al [54] | 2022 | Epoc, Emotiv | Bluetooth, BLE, proprietary | 14 | Semi-dry (saline) | Headset | 128 | 4 | Consumer-grade | | BCI technology |
| Jadavji et al [54] | 2022 | intendiX, g.tec (BCI system) | Bluetooth | 8 | Gel | Headset | 256 | 2 | Medical device (FDA-cleared) | | BCI technology |
| Liao et al [55] | 2022 | Epoc, Emotiv | Bluetooth, BLE, proprietary | 14 | Semi-dry (saline) | Headset | NR | 4 | Consumer-grade | | Biomarker-driven clinical assessment and diagnosis |
| Ramirez-Melendez et al [56] | 2022 | Epoc, Emotiv | Bluetooth, BLE, proprietary | 14 | Semi-dry (saline) | Headset | 128 | 4 | Consumer-grade | | Intervention effect evaluation |
| Westwood et al [57] | 2022 | g.Nautilus, g.tec | Bluetooth, proprietary | 8 | Dry | Cap | 500 | 3 | Medical device (FDA-cleared) | | Intervention effect evaluation |
| Dakwar-Kawar et al [58] | 2023 | g.Nautilus, g.tec | Bluetooth, proprietary | 32 | Gel | Cap | 250 | 3 | Medical device (FDA-cleared) | | Intervention effect evaluation |
| Fan et al [59] | 2023 | Wireless EEG developed by Artise Biomedical | Bluetooth | 32 | Gel | Cap | 500 | 3 | Research use only | | Biomarker-driven clinical assessment and diagnosis |
| Kawai et al [62] | 2023 | Siesta, Compumedics Neuroscan | Wi-Fi | 4 | Gel | Cap | 1024 | 2 | Medical device (FDA-cleared) | | Biomarker-driven clinical assessment and diagnosis |
| Lim et al [60] | 2023 | Not specified | Bluetooth | 2 | Dry | Headband | NR | 4 | Research use only | | BCI technology |
| Luo et al [85] | 2023 | Not specified | NR | 1 | NR | NR | NR | NR | Research use only | | BCI technology |
| Lyu et al [61] | 2023 | FocusCalm, BrainCo | Wi-Fi, Bluetooth | 5 | Dry | Headband | 160 | 4 | Consumer-grade | | BCI technology |
| McCabe et al [63] | 2023 | Mindwave, Neurosky | Bluetooth, BLE | 1 | Dry | Headset | 512 | 4 | Consumer-grade | | Biomarker-driven clinical assessment and diagnosis |
| Shehan et al [64] | 2023 | Insight, Emotiv | Bluetooth, BLE | 5 | Dry | Headset | 128 | 4 | Consumer-grade | | Biomarker-driven clinical assessment and diagnosis |
| Chen et al [65] | 2024 | BR 8, The Brain Research Center of National Chiao Tung University | Bluetooth | 8 | Semi-dry (saline) | Headset | 1000 | 3 | Research use only | | Biomarker-driven clinical assessment and diagnosis |
| Chen et al [96] | 2024 | BR 8, The Brain Research Center of National Chiao Tung University | Bluetooth | 8 | Semi-dry (saline) | Headset | 1000 | 3 | Research use only | | Biomarker-driven clinical assessment and diagnosis |
| Handayani et al [66] | 2024 | Epoc, Emotiv | Bluetooth, BLE, proprietary | 14 | Semi-dry (saline) | Headset | 128 | 4 | Consumer-grade | | Biomarker-driven clinical assessment and diagnosis |
| Huang et al [67] | 2024 | BR 8, The Brain Research Center of National Chiao Tung University | Bluetooth | 8 | Semi-dry (saline) | Headset | 1000 | 3 | Research use only | | Biomarker-driven clinical assessment and diagnosis |
| Kaiser et al [86] | 2024 | Mensia Koala, Mensia Technologies | NR | 8 | Gel | Cap | 512 | 3 | Medical device (CE certificated) | | BCI technology |
| Kwon et al [87] | 2024 | OmniCNS, Omnifit | Bluetooth, BLE | 2 | Dry | Headband | 250 | 4 | Medical device (Korean MFDS-cleared) | | BCI technology |
| Lin et al [68] | 2024 | Wireless EEG developed by Artise Biomedical | Bluetooth | 32 | Semi-dry (saline) | Cap | 1000 | 3 | Research use only | | Biomarker-driven clinical assessment and diagnosis |
| Rebollo et al [89] | 2024 | BrainLink, Macrotellect | Bluetooth, BLE | 2 | Dry | Headset | 512 | 4 | Consumer-grade | | Intervention effect evaluation |
| Tseng et al [69] | 2024 | Modified version of Quick-20, CGX | BLE | 12 | Dry | Headset | 500 | 3 | Medical device (FDA-cleared) | | Biomarker-driven clinical assessment and diagnosis |
| Tseng et al [70] | 2024 | Modified version of Quick-20, CGX | BLE | 12 | Dry | Headset | 500 | 3 | Medical device (FDA-cleared) | | Biomarker-driven clinical assessment and diagnosis |
| Wang et al [71] | 2024 | Focus1, BrainCo | Wi-Fi, Bluetooth | 1 | Gel | Headband | 160 | 4 | Consumer-grade | | BCI technology |
| Cai et al [72] | 2025 | Epoc, Emotiv | Bluetooth, BLE, proprietary | 14 | Semi-dry (saline) | Headset | NR | 4 | Consumer-grade | | Biomarker-driven clinical assessment and diagnosis |
| Huang et al [88] | 2025 | BR 8, The Brain Research Center of National Chiao Tung University | Bluetooth | 8 | Semi-dry (saline) | Headset | 1000 | 3 | Research use only | | Biomarker-driven clinical assessment and diagnosis |
| Kim et al [95] | 2025 | OmniCNS, Omnifit | Bluetooth, BLE | 2 | Dry | Headband | 250 | 4 | Medical device (Korean MFDS-cleared) | | BCI technology |
| Mandapati and Ranjan [73] | 2025 | Epoc, Emotiv | Bluetooth, BLE, proprietary | 14 | Semi-dry (saline) | Headset | 128 | 4 | Consumer-grade | | Intervention effect evaluation |
| Mercado-Aguirre et al [91] | 2025 | Epoc, Emotiv | Bluetooth, BLE, proprietary | 14 | Semi-dry (saline) | Headset | 128 | 4 | Consumer-grade | | Biomarker-driven clinical assessment and diagnosis |
| Pang et al [90] | 2025 | Custom Wireless EEG | NR | Not reported in paper | NR | NR | NR | NR | Research use only | | Biomarker-driven clinical assessment and diagnosis |
| Penmetsa et al [92] | 2025 | MUSE 2, InteraXon | Bluetooth | 4 | Dry | Headband | 256 | 4 | Consumer-grade | | Biomarker-driven clinical assessment and diagnosis |
| Wang et al [93] | 2025 | NeuSen W, Neuracle | Wi-Fi | 32 | Semi-dry (saline) | Cap | 1000 | 3 | Research use only | | Biomarker-driven clinical assessment and diagnosis |
| Dakwar-Kawar et al [74] | 2026 | g.Nautilus, g.tec | Bluetooth, proprietary | 32 | Gel | Cap | 250 | 3 | Medical device (FDA-cleared) | | Intervention effect evaluation |
| Ezedinma et al [94] | 2026 | Nox-A1, Nox Medical | Bluetooth | 3 | Dry | NR | 256 | 2 | Medical device (FDA-cleared) | | Biomarker-driven clinical assessment and diagnosis |

^a^CoME-D score ranges from 0 (off-body) to 5 (head-mounted with no additional equipment).

^b^Devices were classified by regulatory status into three categories: medical devices (cleared or certified by a national regulatory body, including the U.S. FDA, European CE marking, or Korean MFDS), consumer-grade devices (commercially available products not classified as medical devices), and research-use-only (RUO) devices (intended solely for investigational purposes). Previously discontinued devices were reclassified according to their regulatory status at the time of the study.

^c^Application purpose categories: Biomarker-driven clinical assessment and diagnosis; BCI technology (including neurofeedback); Intervention effect evaluation; Task/state monitoring.

**Abbreviations**

BCI: brain–computer interface; BLE: Bluetooth low energy; CE: Conformité Européenne; CoME-D: Categorization of Mobile EEG Devices; EEG: electroencephalography; EPOC: Emotiv EPOC series (includes EPOC, EPOC+, and EPOC-X); FDA: Food and Drug Administration (United States); MFDS: Ministry of Food and Drug Safety (South Korea); NR: not reported; RUO: research use only; Wi-Fi: wireless fidelity.

**References**

33. Modarres-Zadeh M, Xu X, Jaromczyk A, Senders S. A neuro-behavioral test and algorithms for screening and evaluating therapy in ADHD. Conf Proc IEEE Eng Med Biol Soc. 2005;2005:2486-2489. [doi: 10.1109/IEMBS.2005.1616973]

34. Iramina K, Matsuda K, Ide J, Noguchi Y. Monitoring system of neuronal activity and moving activity without restraint using wireless EEG, NIRS and accelerometer. Presented at: 2010 IEEE EMBS Conference on Biomedical Engineering and Sciences (IECBES); Nov 30 to Dec 2, 2010; Kuala Lumpur, Malaysia. 2010. [doi: 10.1109/IECBES.2010.5742285]

35. Israsena P, Hemrungrojn S, Sukwattanasinit N, Maes M. Development and evaluation of an interactive electro-encephalogram-based neurofeedback system for training attention and attention defects in children. J Med Imaging Hlth Inform. Sep 1, 2015;5(5):1045-1052. [doi: 10.1166/jmihi.2015.1492]

36. Zennifa F, Ide J, Noguchi Y, Iramina K. Monitoring of cognitive state on mental retardation child using EEG, ECG and NIRS in four years study. Presented at: 2015 37th Annual International Conference of the IEEE Engineering in Medicine and Biology Society (EMBC); Nov 5, 2015; Milan, Italy. 2015. [doi: 10.1109/EMBC.2015.7319908]

37. Billeci L, Tonacci A, Tartarisco G, et al. An integrated approach for the monitoring of brain and autonomic response of children with autism spectrum disorders during treatment by wearable technologies. Front Neurosci. 2016;10(276):276. [doi: 10.3389/fnins.2016.00276] [Medline: 27445652]

38. Martínez F, Barraza C, González N, González J. KAPEAN: understanding affective states of children with ADHD. J Educ Technol Soc. 2016;19(2):18-28. URL: https://www.jstor.org/stable/jeductechsoci.19.2.18

39. Perales F, Amengual E. Combining EEG and serious games for attention assessment of children with cerebral palsy. Converging Clinical and Engineering Research on Neurorehabilitation II. Biosystems & Biorobotics, vol 15. Cham: Springer; 2017. [doi: 10.1007/978-3-319-46669-9_66]

40. Askari E, Setarehdan SK, Sheikhani A, Mohammadi MR, Teshnehlab M. Modeling the connections of brain regions in children with autism using cellular neural networks and electroencephalography analysis. Artif Intell Med. Jul 2018;89:40-50. [doi: 10.1016/j.artmed.2018.05.003] [Medline: 30007788]

41. Eroglu G, Aydin S, Cetin M, Balcisoy S. Improving cognitive functions of dyslexies using multi-sensory learning and EEG neurofeedback. Presented at: 2018 26th Signal Processing and Communications Applications Conference (SIU); May 2-5, 2018; Izmir. 2018. [doi: 10.1109/SIU.2018.8404711]

42. Mercado-Aguirre IM, Gutiérrez-Ruiz K, Contreras-ortiz SH. Acquisition and analysis of cognitive evoked potentials using an emotiv headset for ADHD evaluation in children. Presented at: 2019 XXII Symposium on Image, Signal Processing and Artificial Vision (STSIVA); Apr 24-26, 2019; Bucaramanga, Colombia. 2019. [doi: 10.1109/STSIVA.2019.8730225]

43. Chu KC, Lu HK, Huang MC, et al. Using mobile electroencephalography and actigraphy to diagnose attention-deficit/hyperactivity disorder: case-control comparison study. JMIR Ment Health. Jun 19, 2020;7(6):e12158. [doi: 10.2196/12158] [Medline: 32558658]

44. Liao CY, Chen RC, Tai SK. Children with attention deficit hyperactivity disorder and learning disability detection via brainwave measurements. ICIC Express Lett B: Appl. 2020;11:51-58. [doi: 10.24507/icicelb.11.01.5]

45. Matamoros OM, Escobar JJM, Tejeida Padilla R, Lina Reyes I. Neurodynamics of patients during a dolphin-assisted therapy by means of a fractal intraneural analysis. Brain Sci. Jun 25, 2020;10(6):403. [doi: 10.3390/brainsci10060403] [Medline: 32630512]

46. Chen IC, Chang CH, Chang Y, Lin DS, Lin CH, Ko LW. Neural dynamics for facilitating ADHD diagnosis in preschoolers: central and parietal delta synchronization in the Kiddie continuous performance test. IEEE Trans Neural Syst Rehabil Eng. 2021;29(1524–33):1524-1533. [doi: 10.1109/TNSRE.2021.3097551] [Medline: 34280103]

47. Peng S, Xu R, Yi X, Hu X, Liu L, Liu L. Early screening of children with autism spectrum disorder based on electroencephalogram signal feature selection with L1-Norm regularization. Front Hum Neurosci. 2021;15:656578. [doi: 10.3389/fnhum.2021.656578] [Medline: 34239427]

48. Serrano-Barroso A, Siugzdaite R, Guerrero-Cubero J, et al. Detecting attention levels in ADHD children with a video game and the measurement of brain activity with a single-channel BCI headset. Sensors (Basel). May 6, 2021;21(9):3221. [doi: 10.3390/s21093221] [Medline: 34066492]

49. Bach-Morrow L, Boccalatte F, DeRosa A, et al. Functional changes in prefrontal cortex following frequency-specific training. Sci Rep. Nov 24, 2022;12(1):20316. [doi: 10.1038/s41598-022-24088-7] [Medline: 36434008]

50. Chang Y, Stevenson C, Chen IC, Lin DS, Ko LW. Neurological state changes indicative of ADHD in children learned via EEG-based LSTM networks. J Neural Eng. Feb 10, 2022;19(1). [doi: 10.1088/1741-2552/ac4f07] [Medline: 35081524]

51. Chen IC, Lee PW, Wang LJ, Chang CH, Lin CH, Ko LW. Incremental validity of multi-method and multi-informant evaluations in the clinical diagnosis of preschool ADHD. J Atten Disord. Aug 2022;26(10):1293-1303. [doi: 10.1177/10870547211045739] [Medline: 34949123]

52. Chen IC, Chen CL, Chang CH, et al. Task-rate-related neural dynamics using wireless EEG to assist diagnosis and intervention planning for preschoolers with ADHD exhibiting heterogeneous cognitive proficiency. J Pers Med. Apr 30, 2022;12(5):731. [doi: 10.3390/jpm12050731] [Medline: 35629153]

53. Giannadou A, Jones M, Freeth M, Samson AC, Milne E. Investigating neural dynamics in autism spectrum conditions outside of the laboratory using mobile electroencephalography. Psychophysiology. Apr 2022;59(4):e13995. [doi: 10.1111/psyp.13995] [Medline: 34982474]

54. Jadavji Z, Zewdie E, Kelly D, Kinney-Lang E, Robu I, Kirton A. Establishing a clinical brain-computer interface program for children with severe neurological disabilities. Cureus. Jun 2022;14(6):e26215. [doi: 10.7759/cureus.26215] [Medline: 35891842]

55. Liao M, Duan H, Wang G. Application of machine learning techniques to detect the children with autism spectrum disorder. J Healthc Eng. 2022;2022(1):9340027. [doi: 10.1155/2022/9340027] [Medline: 35368925]

56. Ramirez-Melendez R, Matamoros E, Hernandez D, Mirabel J, Sanchez E, Escude N. Music-enhanced emotion identification of facial emotions in autistic spectrum disorder children: a pilot EEG study. Brain Sci. May 30, 2022;12(6):704. [doi: 10.3390/brainsci12060704] [Medline: 35741590]

57. Westwood SJ, Bozhilova N, Criaud M, et al. The effect of transcranial direct current stimulation (tDCS) combined with cognitive training on EEG spectral power in adolescent boys with ADHD: a double-blind, randomized, sham-controlled trial. IBRO Neurosci Rep. Jun 2022;12:55-64. [doi: 10.1016/j.ibneur.2021.12.005] [Medline: 35746969]

58. Dakwar-Kawar O, Mairon N, Hochman S, Berger I, Cohen Kadosh R, Nahum M. Transcranial random noise stimulation combined with cognitive training for treating ADHD: a randomized, sham-controlled clinical trial. Transl Psychiatry. Aug 2, 2023;13(1):271. [doi: 10.1038/s41398-023-02547-7] [Medline: 37528107]

59. Fan ZC, Lin RW, Tsai CS, et al. Unveiling multivariate EEG features: a novel approach to enhancing ADHD diagnosis through visual and auditory attention tests. Presented at: 2023 International Conference on Fuzzy Theory and Its Applications (iFUZZY); Oct 26-29, 2023; Penghu, Taiwan. 2023. [doi: 10.1109/iFUZZY60076.2023.10324282]

60. Lim CG, Soh CP, Lim SSY, Fung DSS, Guan C, Lee TS. Home-based brain-computer interface attention training program for attention deficit hyperactivity disorder: a feasibility trial. Child Adolesc Psychiatry Ment Health. Jan 25, 2023;17(1):15. [doi: 10.1186/s13034-022-00539-x] [Medline: 36698168]

61. Lyu Y, An P, Xiao Y, et al. Eggly: designing mobile augmented reality neurofeedback training games for children with autism spectrum disorder. Proc ACM Interact Mob Wearable Ubiquitous Technol. 2023;7:1-29. [doi: 10.1145/3596251]

62. Kawai M, Buck C, Chick CF, et al. Sleep architecture is associated with core symptom severity in autism spectrum disorder. Sleep. Mar 9, 2023;46(3):zsac273. [doi: 10.1093/sleep/zsac273] [Medline: 36385326]

63. McCabe LE, Johnstone SJ, Jiang H, Sun L, Zhang DW. Links between excessive daytime sleepiness and EEG power and activation in two subtypes of ADHD. Biol Psychol. Feb 2023;177:108504. [doi: 10.1016/j.biopsycho.2023.108504] [Medline: 36681294]

64. Shehan A, Anjali S, Sanjula L, Lavanya M, Thelijjagoda S, Krishara J. Neuroassist ADHD analyzer: a smart application for recognizing attention deficit hyperactivity disorder (ADHD) levels in children. Presented at: 2023 International Conference on Innovative Computing, Intelligent Communication and Smart Electrical Systems (ICSES); Dec 14-15, 2023; Chennai, India. 2023. [doi: 10.1109/ICSES60034.2023.10465399]

65. Chen IC, Chang CL, Huang IW, Chang MH, Ko LW. Electrophysiological functional connectivity and complexity reflecting cognitive processing speed heterogeneity in young children with ADHD. Psychiatry Res. Oct 2024;340:116100. [doi: 10.1016/j.psychres.2024.116100] [Medline: 39121760]

66. Handayani N, Asyrafi H, Khotimah SN. EEG complexity in children with autism spectrum disorders: a multiscale entropy analysis. AIP Conf Proc. Aug 28, 2024;3210(1). [doi: 10.1063/5.0228307]

67. Huang IW, Jheng YC, Chen IC, Ko LW. Complexity analysis based on parietal fuzzy entropy to facilitate ADHD diagnosis in young children. Presented at: Annu Int Conf IEEE Eng Med Biol Soc; Jul 15-19, 2024; Orlando, FL, USA. 2024. [doi: 10.1109/EMBC53108.2024.10781919]

68. Lin JW, Fan ZC, Tzou SC, Wang LJ, Ko LW. Temporal alpha dissimilarity of ADHD brain network in comparison with CPT and CATA. IEEE Trans Neural Syst Rehabil Eng. 2024;32:1333-1343. [doi: 10.1109/TNSRE.2024.3360137] [Medline: 38289841]

69. Tseng YL, Lee CH, Chiu YN, et al. Characterizing autism spectrum disorder through fusion of local cortical activation and global functional connectivity using game-based stimuli and a mobile EEG system. IEEE Trans Neural Syst Rehabil Eng. 2024;32:3026-3035. [doi: 10.1109/TNSRE.2024.3417210] [Medline: 39163173]

70. Tseng YL, Liu HH, Chiu YN, et al. Electroencephalography connectivity assesses cognitive disorders of autistic children during game-based social interaction. IEEE Trans Cogn Dev Syst. 2024;16(2):782-793. [doi: 10.1109/TCDS.2023.3297609]

71. Wang XN, Zhang T, Han BC, et al. Wearable EEG neurofeedback based-on machine learning algorithms for children with autism: a randomized, placebo-controlled study. Curr Med Sci. Dec 2024;44(6):1141-1147. [doi: 10.1007/s11596-024-2938-3]

72. Cai C, Wang J, Lin J, et al. Functional connectivity analysis of children with autism under emotional clips. IEEE Trans Affective Comput. 2025;16(3):1646-1659. [doi: 10.1109/TAFFC.2025.3528920]

73. Mandapati M, Ranjan P. Virtual reality based audio visual brainwave entrainment to improve learning in children with attention deficit hyperactive disorder. Appl Neuropsychol Child. Jan 23, 2025:1-15. [doi: 10.1080/21622965.2025.2455102] [Medline: 39847472]

74. Dakwar-Kawar O, Francis AJA, Arya R, et al. The effects of transcranial random noise stimulation on excitation/inhibition balance in ADHD. Neuroimage Clin. 2026;49:103923. [doi: 10.1016/j.nicl.2025.103923] [Medline: 41496381] [Epub 2025 Dec 5]

75. Lim CG, Lee TS, Guan C, et al. A brain-computer interface based attention training program for treating attention deficit hyperactivity disorder. PLoS One. 2012;7(10):e46692. [doi: 10.1371/journal.pone.0046692] [Medline: 23115630]

76. Jiang H, Johnstone SJ. A preliminary multiple case report of neurocognitive training for children with AD/HD in China. Sage Open. Apr 1, 2015;5(2):2158244015586811. [doi: 10.1177/2158244015586811]

77. Garzotto F, Gelsomini M, Pappalardo A, Sanna C, Stella E, Zanella M. Using brain signals in adaptive smart spaces for disabled children. Presented at: Proceedings of the 2016 CHI Conference Extended Abstracts on Human Factors in Computing Systems; May 7-12, 2016; San Jose, California, USA. 2016. [doi: 10.1145/2851581.2892533]

78. Qian X, Loo BRY, Castellanos FX, et al. Brain-computer-interface-based intervention re-normalizes brain functional network topology in children with attention deficit/hyperactivity disorder. Transl Psychiatry. Aug 10, 2018;8(1):149. [doi: 10.1038/s41398-018-0213-8] [Medline: 30097579]

79. Fan J, Wade JW, Key AP, Warren ZE, Sarkar N. EEG-based affect and workload recognition in a virtual driving environment for ASD intervention. IEEE Trans Biomed Eng. Jan 2018;65(1):43-51. [doi: 10.1109/TBME.2017.2693157] [Medline: 28422647]

80. Mercado J, Espinosa-Curiel I, Escobedo L, Tentori M. Developing and evaluating a BCI video game for neurofeedback training: the case of autism. Multimed Tools Appl. May 2019;78(10):13675-13712. [doi: 10.1007/s11042-018-6916-2]

81. Lim CG, Poh XWW, Fung SSD, et al. A randomized controlled trial of a brain-computer interface based attention training program for ADHD. PLoS One. 2019;14(5):e0216225. [doi: 10.1371/journal.pone.0216225] [Medline: 31112554]

82. Teo SHJ, Poh XWW, Lee TS, et al. Brain-computer interface based attention and social cognition training programme for children with ASD and co-occurring ADHD: a feasibility trial. Res Autism Spectr Disord. Nov 2021;89:101882. [doi: 10.1016/j.rasd.2021.101882]

83. Johnstone SJ, Parrish L, Jiang H, Zhang DW, Williams V, Li S. Aiding diagnosis of childhood attention-deficit/hyperactivity disorder of the inattentive presentation: discriminant function analysis of multi-domain measures including EEG. Biol Psychol. Apr 2021;161:108080. [doi: 10.1016/j.biopsycho.2021.108080] [Medline: 33744372]

84. Chen IC, Chang CL, Chang MH, Ko LW. Atypical functional connectivity during rest and task-related dynamic alteration in young children with attention deficit hyperactivity disorder: an analysis using the phase-locking value. Psychiatry Clin Neurosci. Jun 2022;76(6):235-245. [doi: 10.1111/pcn.13344] [Medline: 35235255]

85. Luo X, Guo X, Zhao Q, et al. A randomized controlled study of remote computerized cognitive, neurofeedback, and combined training in the treatment of children with attention-deficit/hyperactivity disorder. Eur Child Adolesc Psychiatry. Aug 2023;32(8):1475-1486. [doi: 10.1007/s00787-022-01956-1]

86. Kaiser A, Aggensteiner PM, Blasco Fontecilla H, et al. Limited usefulness of neurocognitive functioning indices as predictive markers for treatment response to methylphenidate or neurofeedback@home in children and adolescents with ADHD. Front Psychiatry. 2024;14:1331004. [doi: 10.3389/fpsyt.2023.1331004]

87. Kwon SY, Seo G, Jang M, et al. The effect of mobile neurofeedback training in children with attention deficit hyperactivity disorder: a randomized controlled trial. Clin Psychopharmacol Neurosci. Feb 29, 2024;22(1):67-78. [doi: 10.9758/cpn.23.1054] [Medline: 38247413]

88. Huang TC, Yang SR, Chiang SE, He C, Lin DS, Ko LW. EEG slow-rate task features with retrieval-augmented generation for ADHD classification in school-age children. Presented at: 2025 International Automatic Control Conference (CACS); Nov 5-8, 2025; Hsinchu, Taiwan. [doi: 10.1109/CACS67552.2025.11288161]

89. Rebollo C, Tonda A, Sorlí C, Remolar I. Brain waves and learning in children with ADHD: impact of educational video games vs traditional methods. Presented at: 21st International Conference on Cognition and Exploratory Learning in Digital Age (CELDA 2024); Oct 26-28, 2024; Zagreb, Croatia. URL: https://eric.ed.gov/?id=ED665389

90. Pang L, Zhao M, Ma C, et al. A multimodal data-driven assessment system for autism spectrum disorder in children: development and pilot validation of a multimodal acquisition platform. Presented at: 2025 47th Annual International Conference of the IEEE Engineering in Medicine and Biology Society (EMBC); Jul 14-18, 2025; Copenhagen, Denmark. [doi: 10.1109/EMBC58623.2025.11254484]

91. Mercado-Aguirre I, Gutiérrez-Ruiz K, Contreras-Ortiz SH. Classification of auditory ERPs for ADHD detection in children. J Med Eng Technol. Apr 2025;49(3):69-78. [doi: 10.1080/03091902.2025.2477506] [Medline: 40117461]

92. Penmetsa H, Abbasi R, Yellamilli N, et al. Investigating brain activity of children with autism spectrum disorder during STEM-related cognitive tasks. Information. 2025;16(10):880. [doi: 10.3390/info16100880]

93. Wang W, Liu Y, Shi P, et al. Altered tactile abnormalities in children with ASD during tactile processing and recognition revealed by dynamic EEG features. Front Psychiatry. 2025;16:1611438. [doi: 10.3389/fpsyt.2025.1611438] [Medline: 41035957]

94. Ezedinma U, Burgess S, Greenhill J, et al. Home polysomnography in children with autism spectrum disorder: a prospective observational study. J Sleep Res. Aug 2026;35(4):e70265. [doi: 10.1111/jsr.70265] [Medline: 41392642] [Epub 2025 Dec 15]

95. Kim JW, Kim BN, Kim JI, Yang CM, Kwon J. Effects of mobile neurofeedback on theta–gamma coupling and clinical outcomes in children with ADHD: a double-blind, sham-controlled randomized clinical trial. Neuropsychiatr Dis Treat. 2025;21:2733-2746. [doi: 10.2147/NDT.S554756] [Medline: 41368620]

96. Chen IC, Chang CL, Chang MH, Ko LW. The utility of wearable electroencephalography combined with behavioral measures to establish a practical multi-domain model for facilitating the diagnosis of young children with attention-deficit/hyperactivity disorder. J Neurodev Disord. Nov 11, 2024;16(1):62. [doi: 10.1186/s11689-024-09578-1] [Medline: 39528958]
