# Supplementary material for: Wireless Electroencephalography in Research on Children With Developmental Disabilities: Scoping Review
Source: J Med Internet Res. 2026 Aug 25;28:e84707. doi: 10.2196/84707 (PMC13505603; doi:10.2196/84707)
Supplement: Supplementary file 4 [file jmir-v28-e84707-s004.docx]

**Table S1.** Summary of data quality–related reporting characteristics across included studies (N = 64).

| **Characteristics** | **n (%)** |
| --- | --- |
| **Validation against a gold-standard wired EEG system** | **64 (100%)** |
| Not reported | 55 (85.9) |
| Indirect validationᵃ | 6 (9.4) |
| Direct validationᵇ | 3 (4.7) |
| **Artifact handling methods** | **64 (100%)** |
| Not reported | 8 (12.5) |
| *Reported (limited/opaque method)* |  |
| Proprietary algorithm^c^ | 19 (29.7) |
| Only filtering or manual segment rejection | 13 (20.3) |
| *Reported (adequate/transparent method)* |  |
| Blind source separation (ICA or ASR)^d^ | 19 (29.7) |
| Other methodsᵉ | 5 (7.8) |
| **Reported impedance thresholds** | **64 (100%)** |
| Not reported | 51 (79.7) |
| *Reported* |  |
| <100 kΩ | 10 (15.6) |
| <30 kΩ | 2 (3.1) |
| <20 kΩ | 1 (1.6) |

^a^Indirect validation refers to validation supported by prior literature.

^b^Direct validation refers to direct comparison with a gold-standard EEG system within the study.

^c^The manufacturer-specific, proprietary algorithm handles artifacts using a built-in algorithm, often referred to as a “black box.”

^d^Blind source separation (e.g., independent component analysis or artifact subspace reconstruction) refers to methods that decompose mixed EEG signals into latent components or subspaces, enabling the identification and removal of artifact-related activity while preserving neural signals as much as possible.

^e^Other methods included iterative template matching and suppression (n = 2), recursive sparse Bayesian learning (n = 1), automatic epoch rejection (n = 1), and wavelet transform (n = 1).

**Table S2.** Data quality–related reporting characteristics of individual included studies (N = 64).

| Study [reference number] | Wireless EEG model, Manufacturer | Channel | Scalp coverage | Validation against a gold-standard EEG system | Artifact handling method | Impedance reporting |
| --- | --- | --- | --- | --- | --- | --- |
| Modarres-Zadeh et al [33] | Crystal 16, Cleveland Medical Devices | 8 | Partial (frontal, central, occipital) | No validation reported | NR | NR |
| Iramina et al [34] | Custom Wireless EEG | 2 | Partial (frontal, parietal) | No validation reported | Manual segment rejection | NR |
| Lim et al [75] | Wireless EEG developed by Neurosky | 2 | Partial (prefrontal) | No validation reported | Proprietary algorithm (Neurosky) | NR |
| Israsena et al [35] | Mindwave, Neurosky | 1 | Partial (prefrontal) | No validation reported | Proprietary algorithm (Neurosky) | NR |
| Jiang and Johnstone [76] | Mindwave, Neurosky | 1 | Partial (prefrontal) | No validation reported | Proprietary algorithm (Neurosky) | NR |
| Zennifa et al [36] | Custom Wireless EEG | 2 | Partial (frontal, parietal) | No validation reported | Manual segment rejection | NR |
| Billeci et al [37] | Enobio, Neuroelectrics | 19 | Whole | No validation reported | Manual segment rejection | NR |
| Garzotto et al [77] | Mindwave, Neurosky | 1 | Partial (prefrontal) | No validation reported | Proprietary algorithm (Neurosky) | NR |
| Martínez et al [38] | Epoc, Emotiv | 14 | Partial (frontal, temporal, parietal, occipital) | No validation reported | NR | NR |
| Perales et al [39] | Enobio, Neuroelectrics | 8 | Partial (prefrontal, frontal, central) | No validation reported | Basic filtering only | NR |
| Askari et al [40] | Epoc, Emotiv | 14 | Partial (frontal, temporal, parietal, occipital) | No validation reported | Manual segment rejection | NR |
| Eroğlu et al [41] | Epoc, Emotiv | 14 | Partial (frontal, temporal, parietal, occipital) | No validation reported | During BCI: proprietary algorithm  EEG analysis: Manual segment rejection | NR |
| Fan et al [79] | Epoc, Emotiv | 14 | Partial (frontal, temporal, parietal, occipital) | No validation reported | Automatic epoch rejection | NR |
| Qian et al [78] | Wireless EEG developed by Zeo | 2 | Partial (prefrontal) | No validation reported | Proprietary algorithm (Zeo) | NR |
| Lim et al [81] | BCI dry headband | 2 | Partial (prefrontal) | No validation reported | Proprietary algorithm | NR |
| Mercado et al [80] | BrainLink, Macrotellect | 2 | Partial (frontal) | No validation reported | Proprietary algorithm (BrainLink) | NR |
| Mercado-Aguirre et al [42] | Epoc, Emotiv | 14 | Partial (frontal) | Indirect validation (only citation) | Basic filtering only | NR |
| Chu et al [43] | MindSet, NeuroSky | 1 | Partial (prefrontal) | No validation reported | Proprietary algorithm (Neurosky) | NR |
| Liao et al [44] | Mindwave, Neurosky | 1 | Partial (prefrontal) | No validation reported | NR | NR |
| Matamoros et al [45] | Mindwave, Neurosky | 1 | Partial (prefrontal) | No validation reported | Proprietary algorithm (Neurosky) | NR |
| Chen et al [46] | BR 8, National Chiao Tung University | 8 | Partial (prefrontal, frontal, central, parietal, occipital) | Indirect validation (only citation) | BSS (ICA) | < 100 kΩ |
| Johnstone et al [83] | Mindwave, Neurosky | 1 | Partial (prefrontal) | Indirect validation (only citation) | ITMS, Iterative Template Matching and Suppression | NR |
| Peng et al [47] | Epoc, Emotiv | 14 | Partial (frontal, temporal, parietal, occipital) | No validation reported | BSS (ICA) | NR |
| Serrano-Barroso et al [48] | Mindwave, Neurosky | 1 | Partial (prefrontal) | No validation reported | Proprietary algorithm (Neurosky) | NR |
| Teo et al [82] | BCI dry headband | 2 | Partial (prefrontal) | No validation reported | Proprietary algorithm | NR |
| Bach-Morrow et al [49] | THINK headset, Think Interfaces | 2 | Partial (central) | Direct validation (head-to-head comparison with Ag/AgCl electrodes) | NR | NR |
| Chang et al [50] | BR 8, National Chiao Tung University | 8 | Partial (prefrontal, frontal, central, parietal, occipital) | No validation reported | BSS (ASR) | NR |
| Chen et al [51] | BR 8, National Chiao Tung University | 8 | Partial (prefrontal, frontal, central, parietal, occipital) | No validation reported | Manual segment rejection | < 100 kΩ |
| Chen et al [52] | BR 8, National Chiao Tung University | 8 | Partial (prefrontal, frontal, central, parietal, occipital) | Indirect validation (only citation) | BSS (ICA) | < 100 kΩ |
| Chen et al [84] | BR 8, National Chiao Tung University | 8 | Partial (prefrontal, frontal, central, parietal, occipital) | No validation reported | BSS (ICA) | < 100 kΩ |
| Giannadou et al [53] | Eego™ sports, ANTneuro | 32 | Whole | Direct validation (objective data quality validation against lab-based EEG) | BSS (ICA) +Manual segment rejection | < 20 kΩ |
| Jadavji et al [54] | 1. Epoc, Emotiv  2. intendiX, g.tec (BCI)  3. mindBEAGLE, g.tec (BCI) | 14,8,  16 | Partial/partial/partial | No validation reported | Basic filtering only | NR |
| Liao et al [55] | Epoc, Emotiv | 14 | Partial (frontal, temporal, parietal, occipital) | No validation reported | Manual segment rejection | NR |
| Ramirez-Melendez et al [56] | Epoc, Emotiv | 14 | Partial (frontal, temporal, parietal, occipital) | No validation reported | Manual segment rejection | NR |
| Westwood et al [57] | g.Nautilus, g.tec | 8 | Partial (prefrontal, frontal, central, parietal) | No validation reported | BSS (ICA) | < 30 kΩ |
| Dakwar-Kawar et al [58] | g.Nautilus, g.tec | 64 | Partial (frontal) | No validation reported | BSS (ICA) | < 30 kΩ |
| Fan et al [59] | Wireless EEG developed by Artise Biomedical | 32 | Whole | No validation reported | NR | < 100 kΩ |
| Kawai et al [62] | Siesta, Compumedics Neuroscan | 4 | Partial (central, occipital) | No validation reported | NR | NR |
| Lim et al [60] | BCI dry headband | 2 | Partial (prefrontal) | Indirect validation (only citation) | Proprietary algorithm (not specified) | NR |
| Luo et al [85] | Not specified | 1 | Partial (frontal) | No validation reported | Proprietary algorithm | NR |
| Lyu et al [61] | FocusCalm, BrainCo | 5 | Partial (prefrontal) | No validation reported | Proprietary algorithm (BrainCo) | NR |
| McCabe et al [63] | Mindwave, Neurosky | 1 | Partial (prefrontal) | Direct validation (biomarker replication validation) | ITMS, Iterative Template Matching and Suppression | NR |
| Shehan et al [64] | Insight, Emotiv | 5 | Partial (frontal, temporal, parietal) | No validation reported | NR | NR |
| Chen et al [65] | BR 8, National Chiao Tung University | 8 | Partial (prefrontal, frontal, central, parietal, occipital) | No validation reported | BSS (ICA) | < 100 kΩ |
| Chen et al [96] | BR 8, National Chiao Tung University | 8 | Partial (prefrontal, frontal, central, parietal, occipital) | Indirect validation (only citation) | BSS (ICA) | < 100 kΩ |
| Handayani et al [66] | Epoc, Emotiv | 14 | Partial (frontal, temporal, parietal, occipital) | No validation reported | BSS (ICA) | NR |
| Huang et al [67] | BR 8, National Chiao Tung University | 8 | Partial (prefrontal, frontal, central, parietal, occipital) | No validation reported | BSS (ASR) | < 100 kΩ |
| Kaiser et al [86] | Mensia Koala, Mensia Technologies | 8 | Partial (prefrontal, frontal, central, parietal) | No validation reported | NR | NR |
| Kwon et al [87] | OmniCNS, Omnifit | 2 | Partial (frontal) | No validation reported | Proprietary algorithm (Omnifit) | NR |
| Lin et al [68] | Wireless EEG developed by Artise Biomedical | 32 | Whole | No validation reported | BSS (ICA) | < 100 kΩ |
| Rebollo et al [89] | BrainLink, Macrotellect | 2 | Partial (frontal) | No validation reported | Proprietary algorithm (BrainLink) | NR |
| Tseng et al [69] | Modified version of Quick-20, CGX | 12 | Partial (frontal, central, parietal) | No validation reported | BSS (ICA, ASR) | NR |
| Tseng et al [70] | Modified version of Quick-20, CGX | 12 | Partial (frontal, central, parietal) | No validation reported | BSS (ICA, ASR) | NR |
| Wang et al [71] | Focus1, BrainCo | 1 | Partial (prefrontal) | No validation reported | Proprietary algorithm | NR |
| Cai et al [72] | Epoc, Emotiv | 14 | Partial (frontal, temporal, parietal, occipital) | No validation reported | BSS (ICA) | NR |
| Huang et al [88] | BR 8, National Chiao Tung University | 8 | Partial (prefrontal, frontal, central, parietal, occipital) | No validation reported | BSS (ICA, ASR) | < 100 kΩ |
| Kim et al [95] | OmniCNS, Omnifit | 2 | Partial (prefrontal, frontal) | No validation reported | Basic filtering only | NR |
| Mandapati and Ranjan [73] | Epoc, Emotiv | 14 | Partial (frontal, temporal, parietal, occipital) | No validation reported | Basic filtering only | NR |
| Mercado-Aguirre et al [91] | Epoc, Emotiv | 14 | Partial (frontal) | No validation reported | Wavelet Transform | NR |
| Pang et al [90] | Custom Wireless EEG | NR | Partial (frontal) | No validation reported | Basic filtering only | NR |
| Penmetsa et al [92] | MUSE 2, InteraXon | 4 | Partial (frontal, temporal) | No validation reported | BSS (ICA) | NR |
| Wang et al [93] | NeuSen W, Neuracle | 32 | Whole | No validation reported | BSS (ICA) | NR |
| Dakwar-Kawar et al [74] | g.Nautilus, g.tec | 32 | Whole | No validation reported | Source Imaging Based Method (RSBL, Recursive Sparse Bayesian Learning) | NR |
| Ezedinma et al [94] | Nox-A1, Nox Medical | 3 | Partial (prefrontal, frontal) | No validation reported | Proprietary algorithm (Noxturnal) +Manual segment rejection | NR |

**Abbreviations:** ASR, artifact subspace reconstruction; BCI, brain–computer interface; BSS, blind source separation; EEG, electroencephalography; ICA, independent component analysis; ITMS, iterative template matching and suppression; NR, not reported; RSBL, random subspace binary logic

**References**

33. Modarres-Zadeh M, Xu X, Jaromczyk A, Senders S. A neuro-behavioral test and algorithms for screening and evaluating therapy in ADHD. Conf Proc IEEE Eng Med Biol Soc. 2005;2005:2486-2489. [doi: 10.1109/IEMBS.2005.1616973]

34. Iramina K, Matsuda K, Ide J, Noguchi Y. Monitoring system of neuronal activity and moving activity without restraint using wireless EEG, NIRS and accelerometer. Presented at: 2010 IEEE EMBS Conference on Biomedical Engineering and Sciences (IECBES); Nov 30 to Dec 2, 2010; Kuala Lumpur, Malaysia. 2010. [doi: 10.1109/IECBES.2010.5742285]

35. Israsena P, Hemrungrojn S, Sukwattanasinit N, Maes M. Development and evaluation of an interactive electro-encephalogram-based neurofeedback system for training attention and attention defects in children. J Med Imaging Hlth Inform. Sep 1, 2015;5(5):1045-1052. [doi: 10.1166/jmihi.2015.1492]

36. Zennifa F, Ide J, Noguchi Y, Iramina K. Monitoring of cognitive state on mental retardation child using EEG, ECG and NIRS in four years study. Presented at: 2015 37th Annual International Conference of the IEEE Engineering in Medicine and Biology Society (EMBC); Nov 5, 2015; Milan, Italy. 2015. [doi: 10.1109/EMBC.2015.7319908]

37. Billeci L, Tonacci A, Tartarisco G, et al. An integrated approach for the monitoring of brain and autonomic response of children with autism spectrum disorders during treatment by wearable technologies. Front Neurosci. 2016;10(276):276. [doi: 10.3389/fnins.2016.00276] [Medline: 27445652]

38. Martínez F, Barraza C, González N, González J. KAPEAN: understanding affective states of children with ADHD. J Educ Technol Soc. 2016;19(2):18-28. URL: https://www.jstor.org/stable/jeductechsoci.19.2.18

39. Perales F, Amengual E. Combining EEG and serious games for attention assessment of children with cerebral palsy. Converging Clinical and Engineering Research on Neurorehabilitation II. Biosystems & Biorobotics, vol 15. Cham: Springer; 2017. [doi: 10.1007/978-3-319-46669-9_66]

40. Askari E, Setarehdan SK, Sheikhani A, Mohammadi MR, Teshnehlab M. Modeling the connections of brain regions in children with autism using cellular neural networks and electroencephalography analysis. Artif Intell Med. Jul 2018;89:40-50. [doi: 10.1016/j.artmed.2018.05.003] [Medline: 30007788]

41. Eroglu G, Aydin S, Cetin M, Balcisoy S. Improving cognitive functions of dyslexies using multi-sensory learning and EEG neurofeedback. Presented at: 2018 26th Signal Processing and Communications Applications Conference (SIU); May 2-5, 2018; Izmir. 2018. [doi: 10.1109/SIU.2018.8404711]

42. Mercado-Aguirre IM, Gutiérrez-Ruiz K, Contreras-ortiz SH. Acquisition and analysis of cognitive evoked potentials using an emotiv headset for ADHD evaluation in children. Presented at: 2019 XXII Symposium on Image, Signal Processing and Artificial Vision (STSIVA); Apr 24-26, 2019; Bucaramanga, Colombia. 2019. [doi: 10.1109/STSIVA.2019.8730225]

43. Chu KC, Lu HK, Huang MC, et al. Using mobile electroencephalography and actigraphy to diagnose attention-deficit/hyperactivity disorder: case-control comparison study. JMIR Ment Health. Jun 19, 2020;7(6):e12158. [doi: 10.2196/12158] [Medline: 32558658]

44. Liao CY, Chen RC, Tai SK. Children with attention deficit hyperactivity disorder and learning disability detection via brainwave measurements. ICIC Express Lett B: Appl. 2020;11:51-58. [doi: 10.24507/icicelb.11.01.5]

45. Matamoros OM, Escobar JJM, Tejeida Padilla R, Lina Reyes I. Neurodynamics of patients during a dolphin-assisted therapy by means of a fractal intraneural analysis. Brain Sci. Jun 25, 2020;10(6):403. [doi: 10.3390/brainsci10060403] [Medline: 32630512]

46. Chen IC, Chang CH, Chang Y, Lin DS, Lin CH, Ko LW. Neural dynamics for facilitating ADHD diagnosis in preschoolers: central and parietal delta synchronization in the Kiddie continuous performance test. IEEE Trans Neural Syst Rehabil Eng. 2021;29(1524–33):1524-1533. [doi: 10.1109/TNSRE.2021.3097551] [Medline: 34280103]

47. Peng S, Xu R, Yi X, Hu X, Liu L, Liu L. Early screening of children with autism spectrum disorder based on electroencephalogram signal feature selection with L1-Norm regularization. Front Hum Neurosci. 2021;15:656578. [doi: 10.3389/fnhum.2021.656578] [Medline: 34239427]

48. Serrano-Barroso A, Siugzdaite R, Guerrero-Cubero J, et al. Detecting attention levels in ADHD children with a video game and the measurement of brain activity with a single-channel BCI headset. Sensors (Basel). May 6, 2021;21(9):3221. [doi: 10.3390/s21093221] [Medline: 34066492]

49. Bach-Morrow L, Boccalatte F, DeRosa A, et al. Functional changes in prefrontal cortex following frequency-specific training. Sci Rep. Nov 24, 2022;12(1):20316. [doi: 10.1038/s41598-022-24088-7] [Medline: 36434008]

50. Chang Y, Stevenson C, Chen IC, Lin DS, Ko LW. Neurological state changes indicative of ADHD in children learned via EEG-based LSTM networks. J Neural Eng. Feb 10, 2022;19(1). [doi: 10.1088/1741-2552/ac4f07] [Medline: 35081524]

51. Chen IC, Lee PW, Wang LJ, Chang CH, Lin CH, Ko LW. Incremental validity of multi-method and multi-informant evaluations in the clinical diagnosis of preschool ADHD. J Atten Disord. Aug 2022;26(10):1293-1303. [doi: 10.1177/10870547211045739] [Medline: 34949123]

52. Chen IC, Chen CL, Chang CH, et al. Task-rate-related neural dynamics using wireless EEG to assist diagnosis and intervention planning for preschoolers with ADHD exhibiting heterogeneous cognitive proficiency. J Pers Med. Apr 30, 2022;12(5):731. [doi: 10.3390/jpm12050731] [Medline: 35629153]

53. Giannadou A, Jones M, Freeth M, Samson AC, Milne E. Investigating neural dynamics in autism spectrum conditions outside of the laboratory using mobile electroencephalography. Psychophysiology. Apr 2022;59(4):e13995. [doi: 10.1111/psyp.13995] [Medline: 34982474]

54. Jadavji Z, Zewdie E, Kelly D, Kinney-Lang E, Robu I, Kirton A. Establishing a clinical brain-computer interface program for children with severe neurological disabilities. Cureus. Jun 2022;14(6):e26215. [doi: 10.7759/cureus.26215] [Medline: 35891842]

55. Liao M, Duan H, Wang G. Application of machine learning techniques to detect the children with autism spectrum disorder. J Healthc Eng. 2022;2022(1):9340027. [doi: 10.1155/2022/9340027] [Medline: 35368925]

56. Ramirez-Melendez R, Matamoros E, Hernandez D, Mirabel J, Sanchez E, Escude N. Music-enhanced emotion identification of facial emotions in autistic spectrum disorder children: a pilot EEG study. Brain Sci. May 30, 2022;12(6):704. [doi: 10.3390/brainsci12060704] [Medline: 35741590]

57. Westwood SJ, Bozhilova N, Criaud M, et al. The effect of transcranial direct current stimulation (tDCS) combined with cognitive training on EEG spectral power in adolescent boys with ADHD: a double-blind, randomized, sham-controlled trial. IBRO Neurosci Rep. Jun 2022;12:55-64. [doi: 10.1016/j.ibneur.2021.12.005] [Medline: 35746969]

58. Dakwar-Kawar O, Mairon N, Hochman S, Berger I, Cohen Kadosh R, Nahum M. Transcranial random noise stimulation combined with cognitive training for treating ADHD: a randomized, sham-controlled clinical trial. Transl Psychiatry. Aug 2, 2023;13(1):271. [doi: 10.1038/s41398-023-02547-7] [Medline: 37528107]

59. Fan ZC, Lin RW, Tsai CS, et al. Unveiling multivariate EEG features: a novel approach to enhancing ADHD diagnosis through visual and auditory attention tests. Presented at: 2023 International Conference on Fuzzy Theory and Its Applications (iFUZZY); Oct 26-29, 2023; Penghu, Taiwan. 2023. [doi: 10.1109/iFUZZY60076.2023.10324282]

60. Lim CG, Soh CP, Lim SSY, Fung DSS, Guan C, Lee TS. Home-based brain-computer interface attention training program for attention deficit hyperactivity disorder: a feasibility trial. Child Adolesc Psychiatry Ment Health. Jan 25, 2023;17(1):15. [doi: 10.1186/s13034-022-00539-x] [Medline: 36698168]

61. Lyu Y, An P, Xiao Y, et al. Eggly: designing mobile augmented reality neurofeedback training games for children with autism spectrum disorder. Proc ACM Interact Mob Wearable Ubiquitous Technol. 2023;7:1-29. [doi: 10.1145/3596251]

62. Kawai M, Buck C, Chick CF, et al. Sleep architecture is associated with core symptom severity in autism spectrum disorder. Sleep. Mar 9, 2023;46(3):zsac273. [doi: 10.1093/sleep/zsac273] [Medline: 36385326]

63. McCabe LE, Johnstone SJ, Jiang H, Sun L, Zhang DW. Links between excessive daytime sleepiness and EEG power and activation in two subtypes of ADHD. Biol Psychol. Feb 2023;177:108504. [doi: 10.1016/j.biopsycho.2023.108504] [Medline: 36681294]

64. Shehan A, Anjali S, Sanjula L, Lavanya M, Thelijjagoda S, Krishara J. Neuroassist ADHD analyzer: a smart application for recognizing attention deficit hyperactivity disorder (ADHD) levels in children. Presented at: 2023 International Conference on Innovative Computing, Intelligent Communication and Smart Electrical Systems (ICSES); Dec 14-15, 2023; Chennai, India. 2023. [doi: 10.1109/ICSES60034.2023.10465399]

65. Chen IC, Chang CL, Huang IW, Chang MH, Ko LW. Electrophysiological functional connectivity and complexity reflecting cognitive processing speed heterogeneity in young children with ADHD. Psychiatry Res. Oct 2024;340:116100. [doi: 10.1016/j.psychres.2024.116100] [Medline: 39121760]

66. Handayani N, Asyrafi H, Khotimah SN. EEG complexity in children with autism spectrum disorders: a multiscale entropy analysis. AIP Conf Proc. Aug 28, 2024;3210(1). [doi: 10.1063/5.0228307]

67. Huang IW, Jheng YC, Chen IC, Ko LW. Complexity analysis based on parietal fuzzy entropy to facilitate ADHD diagnosis in young children. Presented at: Annu Int Conf IEEE Eng Med Biol Soc; Jul 15-19, 2024; Orlando, FL, USA. 2024. [doi: 10.1109/EMBC53108.2024.10781919]

68. Lin JW, Fan ZC, Tzou SC, Wang LJ, Ko LW. Temporal alpha dissimilarity of ADHD brain network in comparison with CPT and CATA. IEEE Trans Neural Syst Rehabil Eng. 2024;32:1333-1343. [doi: 10.1109/TNSRE.2024.3360137] [Medline: 38289841]

69. Tseng YL, Lee CH, Chiu YN, et al. Characterizing autism spectrum disorder through fusion of local cortical activation and global functional connectivity using game-based stimuli and a mobile EEG system. IEEE Trans Neural Syst Rehabil Eng. 2024;32:3026-3035. [doi: 10.1109/TNSRE.2024.3417210] [Medline: 39163173]

70. Tseng YL, Liu HH, Chiu YN, et al. Electroencephalography connectivity assesses cognitive disorders of autistic children during game-based social interaction. IEEE Trans Cogn Dev Syst. 2024;16(2):782-793. [doi: 10.1109/TCDS.2023.3297609]

71. Wang XN, Zhang T, Han BC, et al. Wearable EEG neurofeedback based-on machine learning algorithms for children with autism: a randomized, placebo-controlled study. Curr Med Sci. Dec 2024;44(6):1141-1147. [doi: 10.1007/s11596-024-2938-3]

72. Cai C, Wang J, Lin J, et al. Functional connectivity analysis of children with autism under emotional clips. IEEE Trans Affective Comput. 2025;16(3):1646-1659. [doi: 10.1109/TAFFC.2025.3528920]

73. Mandapati M, Ranjan P. Virtual reality based audio visual brainwave entrainment to improve learning in children with attention deficit hyperactive disorder. Appl Neuropsychol Child. Jan 23, 2025:1-15. [doi: 10.1080/21622965.2025.2455102] [Medline: 39847472]

74. Dakwar-Kawar O, Francis AJA, Arya R, et al. The effects of transcranial random noise stimulation on excitation/inhibition balance in ADHD. Neuroimage Clin. 2026;49:103923. [doi: 10.1016/j.nicl.2025.103923] [Medline: 41496381] [Epub 2025 Dec 5]

75. Lim CG, Lee TS, Guan C, et al. A brain-computer interface based attention training program for treating attention deficit hyperactivity disorder. PLoS One. 2012;7(10):e46692. [doi: 10.1371/journal.pone.0046692] [Medline: 23115630]

76. Jiang H, Johnstone SJ. A preliminary multiple case report of neurocognitive training for children with AD/HD in China. Sage Open. Apr 1, 2015;5(2):2158244015586811. [doi: 10.1177/2158244015586811]

77. Garzotto F, Gelsomini M, Pappalardo A, Sanna C, Stella E, Zanella M. Using brain signals in adaptive smart spaces for disabled children. Presented at: Proceedings of the 2016 CHI Conference Extended Abstracts on Human Factors in Computing Systems; May 7-12, 2016; San Jose, California, USA. 2016. [doi: 10.1145/2851581.2892533]

78. Qian X, Loo BRY, Castellanos FX, et al. Brain-computer-interface-based intervention re-normalizes brain functional network topology in children with attention deficit/hyperactivity disorder. Transl Psychiatry. Aug 10, 2018;8(1):149. [doi: 10.1038/s41398-018-0213-8] [Medline: 30097579]

79. Fan J, Wade JW, Key AP, Warren ZE, Sarkar N. EEG-based affect and workload recognition in a virtual driving environment for ASD intervention. IEEE Trans Biomed Eng. Jan 2018;65(1):43-51. [doi: 10.1109/TBME.2017.2693157] [Medline: 28422647]

80. Mercado J, Espinosa-Curiel I, Escobedo L, Tentori M. Developing and evaluating a BCI video game for neurofeedback training: the case of autism. Multimed Tools Appl. May 2019;78(10):13675-13712. [doi: 10.1007/s11042-018-6916-2]

81. Lim CG, Poh XWW, Fung SSD, et al. A randomized controlled trial of a brain-computer interface based attention training program for ADHD. PLoS One. 2019;14(5):e0216225. [doi: 10.1371/journal.pone.0216225] [Medline: 31112554]

82. Teo SHJ, Poh XWW, Lee TS, et al. Brain-computer interface based attention and social cognition training programme for children with ASD and co-occurring ADHD: a feasibility trial. Res Autism Spectr Disord. Nov 2021;89:101882. [doi: 10.1016/j.rasd.2021.101882]

83. Johnstone SJ, Parrish L, Jiang H, Zhang DW, Williams V, Li S. Aiding diagnosis of childhood attention-deficit/hyperactivity disorder of the inattentive presentation: discriminant function analysis of multi-domain measures including EEG. Biol Psychol. Apr 2021;161:108080. [doi: 10.1016/j.biopsycho.2021.108080] [Medline: 33744372]

84. Chen IC, Chang CL, Chang MH, Ko LW. Atypical functional connectivity during rest and task-related dynamic alteration in young children with attention deficit hyperactivity disorder: an analysis using the phase-locking value. Psychiatry Clin Neurosci. Jun 2022;76(6):235-245. [doi: 10.1111/pcn.13344] [Medline: 35235255]

85. Luo X, Guo X, Zhao Q, et al. A randomized controlled study of remote computerized cognitive, neurofeedback, and combined training in the treatment of children with attention-deficit/hyperactivity disorder. Eur Child Adolesc Psychiatry. Aug 2023;32(8):1475-1486. [doi: 10.1007/s00787-022-01956-1]

86. Kaiser A, Aggensteiner PM, Blasco Fontecilla H, et al. Limited usefulness of neurocognitive functioning indices as predictive markers for treatment response to methylphenidate or neurofeedback@home in children and adolescents with ADHD. Front Psychiatry. 2024;14:1331004. [doi: 10.3389/fpsyt.2023.1331004]

87. Kwon SY, Seo G, Jang M, et al. The effect of mobile neurofeedback training in children with attention deficit hyperactivity disorder: a randomized controlled trial. Clin Psychopharmacol Neurosci. Feb 29, 2024;22(1):67-78. [doi: 10.9758/cpn.23.1054] [Medline: 38247413]

88. Huang TC, Yang SR, Chiang SE, He C, Lin DS, Ko LW. EEG slow-rate task features with retrieval-augmented generation for ADHD classification in school-age children. Presented at: 2025 International Automatic Control Conference (CACS); Nov 5-8, 2025; Hsinchu, Taiwan. [doi: 10.1109/CACS67552.2025.11288161]

89. Rebollo C, Tonda A, Sorlí C, Remolar I. Brain waves and learning in children with ADHD: impact of educational video games vs traditional methods. Presented at: 21st International Conference on Cognition and Exploratory Learning in Digital Age (CELDA 2024); Oct 26-28, 2024; Zagreb, Croatia. URL: https://eric.ed.gov/?id=ED665389

90. Pang L, Zhao M, Ma C, et al. A multimodal data-driven assessment system for autism spectrum disorder in children: development and pilot validation of a multimodal acquisition platform. Presented at: 2025 47th Annual International Conference of the IEEE Engineering in Medicine and Biology Society (EMBC); Jul 14-18, 2025; Copenhagen, Denmark. [doi: 10.1109/EMBC58623.2025.11254484]

91. Mercado-Aguirre I, Gutiérrez-Ruiz K, Contreras-Ortiz SH. Classification of auditory ERPs for ADHD detection in children. J Med Eng Technol. Apr 2025;49(3):69-78. [doi: 10.1080/03091902.2025.2477506] [Medline: 40117461]

92. Penmetsa H, Abbasi R, Yellamilli N, et al. Investigating brain activity of children with autism spectrum disorder during STEM-related cognitive tasks. Information. 2025;16(10):880. [doi: 10.3390/info16100880]

93. Wang W, Liu Y, Shi P, et al. Altered tactile abnormalities in children with ASD during tactile processing and recognition revealed by dynamic EEG features. Front Psychiatry. 2025;16:1611438. [doi: 10.3389/fpsyt.2025.1611438] [Medline: 41035957]

94. Ezedinma U, Burgess S, Greenhill J, et al. Home polysomnography in children with autism spectrum disorder: a prospective observational study. J Sleep Res. Aug 2026;35(4):e70265. [doi: 10.1111/jsr.70265] [Medline: 41392642] [Epub 2025 Dec 15]

95. Kim JW, Kim BN, Kim JI, Yang CM, Kwon J. Effects of mobile neurofeedback on theta–gamma coupling and clinical outcomes in children with ADHD: a double-blind, sham-controlled randomized clinical trial. Neuropsychiatr Dis Treat. 2025;21:2733-2746. [doi: 10.2147/NDT.S554756] [Medline: 41368620]

96. Chen IC, Chang CL, Chang MH, Ko LW. The utility of wearable electroencephalography combined with behavioral measures to establish a practical multi-domain model for facilitating the diagnosis of young children with attention-deficit/hyperactivity disorder. J Neurodev Disord. Nov 11, 2024;16(1):62. [doi: 10.1186/s11689-024-09578-1] [Medline: 39528958]
